# Metataxonomic profiling of microbial communities and metabolic analyses of the traditional Spanish raw cow’s milk cheese ‘Casín’ from manufacture to ripening

**DOI:** 10.3389/fmicb.2025.1722502

**Published:** 2025-12-16

**Authors:** Javier Rodríguez, Paula R. Suárez, Lucía Vázquez, Ana Belén Flórez, Ana María Vivar-Quintana, Baltasar Mayo

**Affiliations:** 1 Departamento de Microbiología y Bioquímica, Instituto de Productos Lácteos de Asturias (IPLA), Consejo Superior de Investigaciones Científicas (CSIC), Villaviciosa, Spain; 2 Instituto de Investigación Sanitaria del Principado de Asturias (ISPA), Oviedo, Spain; 3 Área de Tecnología de Alimentos, Escuela Politécnica Superior de Zamora, Universidad de Salamanca, Avenida Requejo, Zamora, Spain

**Keywords:** Casín, traditional cheese, cheese microbiota, metataxonomics, metabolomics, lactic acid bacteria, starters, adjunct cultures

## Abstract

‘Casín’ is a soft, rindless, strongly flavored, Spanish PDO cheese made from raw cow’s milk, the production of which involves a distinctive kneading process during ripening. This study aimed to characterize the bacterial and fungal communities developing during Casín cheese manufacture and ripening, assess their metabolic activity on milk components, and explore correlations between microbial dynamics and physicochemical and volatile organic compounds (VOC) profiles. Bacterial and fungal populations were assessed by culturing and high-throughput amplification and sequencing of 16S rRNA gene and ITS ribosomal regions, while basic tests and high-resolution chromatography techniques were employed to assess physico-chemical parameters and metabolite profiles. Metataxonomic analyses revealed a rich microbial diversity during Casín cheese manufacture and ripening, with a progressive decline in diversity as maturation advanced. Thirty-two bacterial and 30 fungal phylotypes were detected at relative abundances >0.5% in at least one sample. *Lactococcus lactis*/*L. cremoris* and *Geotrichum candidum* consistently dominated bacterial and fungal communities, respectively, across all batches and time points. Concentrations of lactic and butyric acids increased throughout ripening, reflecting active fermentation and lipolysis. Among the VOC, hexanoic, butanoic, octanoic, and n-decanoic acids were the most abundant, with total VOC levels rising steadily and peaking at day 60. These compounds likely contributed to the strong characteristic aroma of mature Casín cheese. Co-occurrence and co-exclusion network analyses, combined with correlations between microbial and metabolic data, suggested that specific microorganisms play central roles in developing the distinctive sensory characteristics of Casín cheese. The results highlight the pivotal contribution of the microorganisms to the biochemical transformations underlying Casín cheese ripening. Dominant taxa, supplemented or not with subdominant taxa, have the potential to serve as the basis for developing a defined complex starter culture, aiming at maintaining the sensory distinctiveness of this PDO cheese, while improving process consistency and microbial safety.

## 1 Introduction

Microorganisms play a pivotal role during milk fermentation, breaking down milk components to yield metabolites such as lactic acid, free amino acids, fatty acids and volatile compounds, all of which are instrumental in shaping the sensory properties of cheeses (Štefániková et al., 2020; Unno et al., 2021), including texture, taste, and aroma (Rampanti et al., 2022). The cheese microbiota is also important from a safety viewpoint; via a variety of competitive mechanisms, it impedes the colonization and proliferation of pathogens and spoilage microorganisms (Lee et al., 2020; Piqueras et al., 2021), thereby enhancing the food’s quality and shelf life (Nam et al., 2021).

Recent advances in next-generation sequencing (NGS) techniques, coupled with novel metabolomic methodologies and sophisticated bioinformatic tools, have allowed for profound insights into the microbial diversity and biochemical potential of cheese-associated microbiotas (Afshari et al., 2020; Alessandria et al., 2017; Ceugniez et al., 2017a; Ceugniez et al., 2017b; Ercolini et al., 2012; Jin et al., 2018). These techniques afford a comprehensive understanding of diverse microbial populations, both prokaryotic and eukaryotic, and their dynamics in several cheeses, which may well correlate with pivotal chemical biomarkers for taste and aroma (Biolcati et al., 2022; Escobar-Zepeda et al., 2016; Lessard et al., 2014; Levante et al., 2023; Li et al., 2017; Monnet et al., 2016; Rampanti et al., 2022). Knowledge of such microbiological-chemical relationships could help optimize cheese manufacturing and ripening, allowing for improvements in overall cheese quality (Carpino et al., 2017; Yeluri Jonnala et al., 2018).

Casín, a traditional cheese made from raw cowʼs milk in the Principality of Asturias, Northern Spain, has enjoyed Protected Designation of Origin (PDO) status since 2008. According to its Technical Annex^1^, its current manufacture entails a mixed enzymatic and acid coagulation, through the use of calf rennet and starter cultures, of evening and morning milk blends at 30–35 °C in stainless steel vats, followed by cutting the curd into hazelnut-sized grains and draining the whey in cheesecloths for 2–3 days, after which Coarse salt is then applied to the surface of the pieces (Alegría et al., 2009; Supplementary Figure S1). A distinctive feature of Casín manufacture involves a weekly mechanical kneading of the cheese during ripening, resulting the cheese in a crust-less, cylindrical, semi-sphere (12–15 cm diameter, 5–7 cm height). Of note, the flat side of the cheese is then adorned with manufacturer-specific stamps. To develop specific starters and/or adjunct cultures –an unaddressed point in the Technical Annex of Casín cheese – previous studies have characterized this cheese’s bacterial communities throughout manufacture and ripening using culture-dependent and culture-independent techniques, which suggested lactic acid bacteria (LAB) to make up the predominant bacterial populations (Alegría et al., 2009).

This study aims to further assess the microbial diversity and dynamics of Casín cheese via a metataxonomic profiling of the bacterial and fungal populations present over manufacturing and ripening. A comprehensive analysis of the metabolites, mostly of microbial origin, including organic acids and volatile compounds, was also conducted. The microbial and biochemical variables were finally subjected to statistical examination, with the aim of identify correlations between microbial and metabolic profiles. This provided insights with regard to the control of fermentation and ripening, which might be of help to improve final cheese quality.

## 2 Materials and methods

### 2.1 Cheese sampling

One batch of Casín cheese was collected from each of three independent manufacturers (A, B and C). Milk (M), curd (C), and cheeses were sampled according to FIL-IDF standard 50B and transported to the laboratory under refrigerated conditions. Cheeses were sampled at 3, 7, 15, 30 and 60 days after manufacture. All producers employed commercial starters for acidification composed of mixtures of LAB species and strains. Producer A used a starter composed of *Lactococcus lactis* subsp. *lactis* biovar. *diacetylactis*, *Leuconostoc* spp., *Streptococcus thermophilus*, and *Levilactobacillus brevis* (Coquard, Villefranche sur Saône, France); producer B used *L. lactis* (including the biovar. *diacetylactis*), *Lactococcus cremoris*, and *S. thermophilus* as a starter (Danisco, Copenhagen, Denmark); and producer C used a complex starter composed of strains *L. lactis* (including the biovar. *diacetylactis*), *L. cremoris*, and *Leuconostoc* spp. (Flora Danica; Chr. Hansen, Hørsholm, Denmark).

### 2.2 Microbiological analyses

Ten grams of curd and cheese samples from each batch were homogenized with 90 mL of a 2% (w/v) sterilized sodium citrate solution at 45 °C for 1 min in a Stomacher 400 (Cosworth, London, UK). Tenfold dilutions of milk and cheese homogenates were prepared with sterile Ringer’s solution (Merck, Darmstadt, Germany) and plated onto specific media. Total aerobic mesophilic bacteria were counted on Plate Count Milk Agar (PCMA) (Merck) after 24 h of incubation at 32 °C; lactococci were counted on M17 agar (Formedium, Norfolk, UK) supplemented with glucose (0.5%) (GM17A) after 48 h of incubation at 32 °C; lactobacilli were counted on de Man, Rogosa and Sharpe agar (MRSA) (Merck) after 48 h of incubation at 32 °C; enterococci were counted on Slanetz and Bartley agar (SBA) (Merck) after 24 h of incubation at 42 °C; enterobacteria and coliforms were counted on Violet Red Bile Glucose agar (VRBGA) (Merck) and Violet Red Bile Lactose agar (VRBLA) (Merck), respectively, using the pour-plate and overlay technique, and enumerating after 24 h of incubation at 37 °C; micrococci and staphylococci were counted on Baird-Parker agar (BPA) (Merck) supplemented with egg yolk tellurite solution (Biokar Diagnostics, Allonne, France) after 24 h of incubation at 37 °C; and yeasts and molds were counted on Yeast-Extract Glucose Chloramphenicol agar (YGCA) (Merck) after 3–5 days of incubation at 25 °C.

### 2.3 Metataxonomic profiling

Curd and cheese samples (5 g of the core) from each of the batches were independently homogenized in the Stomacher with 45 mL of 2% (w/v) sodium citrate solution at 45 °C. After centrifugation at 10,000 rpm for 10 min at 4 °C, the top fat layer was removed using a sterile cotton swab, the supernatant discarded, and the microbial pellet used for total DNA extraction using the Food-Extract DNA Purification Kit (EURx, Gdańsk, Poland), according to the manufacturer’s instructions, but with the following modifications: the commercial lysis buffer Res FE was supplemented with 20 mg mL^−1^ lysozyme (Merck), 25 U mutanolysin (Sigma-Aldrich, Saint Louis, MO., USA), and 10 μg lysostaphin (Sigma-Aldrich). Cell suspensions were incubated at 37 °C for 45 min, and then at 55 °C for 15 min. Incubated cells were then subjected to mechanical lysis with 0.5–1.0 mm crystal beads (BeadTubeDry; EURx) using a FastPrepFP120 Cell Disrupter (Qbiogene, Carlsbad, CA, USA) at 5.5 m s^−1^ for 30 s. The DNA was then purified using the Food-Extract DNA Purification Kit according to the manufacturer’s recommendations. Finally, DNA was quantified fluorometrically in a Qubit 4.0 fluorometer (Invitrogen, Carlsbad, CA, USA) employing the Qubit 1 X dsDNA BR Assay Kit (Invitrogen). DNA quality was determined by measuring the A260/230 nm and A260/280 nm absorbance ratios using a Genova Bio UV–visible spectrophotometer (Jenway, Staffordshire, UK). Purified DNA was stored until processing at −20 °C.

Segments of ∼445 bp of the prokaryotic 16S rRNA gene (V3-V4 hypervariable region), and ~700 bp of the fungal internal transcribed spacers1 and 2 (ITS1 – ITS2) of the ribosomal region, were independently amplified by PCR and sequenced. These regions were amplified, respectively, with primer pairs 338f (5′-TACGGGAGGCAGCAG-3′) and 806r (5′-GGACTACHVGGGTWTCTAATCC-3′) (Wang and Qian, 2009; Caporaso et al., 2012), and ITS5 (5′-GGAAGTAAAAGTCGTAACAAGG-3′) and NCL2 (5′-GAGCTGCATTCCCAAACAACTC-3′) (White et al., 1990; Martin and Rygiewicz, 2005). All primers included overhang adapter sequences compatible with Illumina sequencing. Amplicons were purified using the GenElute PCR Clean-Up Kit (Sigma-Aldrich) and sequenced using the Illumina platform at Eurofin Genomics (Ebersberg, Germany), selecting the INVIEW Microbiome Profiling 3.0 option (Eurofins Genomics) for metataxonomic analysis. From the raw data, Illumina adapter sequences were removed using the CutAdapt program (Martin, 2011), and the reads were subsequently quality-filtered (Q30) using FASTQ (Chen et al., 2018).

Quality-checked and cleaned read sequences were analyzed using QIIME 2 (v.2023.2^2^) (Bolyen et al., 2019), selecting the options paired-end reads and single-end reads for bacterial and eukaryotic metataxonomic analysis, respectively. These sequences were demultiplexed, denoised (to remove noisy sequences, chimeric sequences and singletons), and merged, when possible, using the QIIME 2 q2-dada2 plugin (Callahan et al., 2016). Amplicon sequence variants (ASVs) were taxonomically assigned (>99% identity) using the QIIME 2 feature-classifier plugin, with the DAIRYdb^3^ (Meola et al., 2019) and UNITE^4^ (Kõljalg et al., 2013) reference databases. Contaminant mitochondrial and chloroplast sequences were removed using the QIIME 2 filter-table and filter-seq scripts of the taxa plugin. Similarly, ASVs with <10 copies for the total of all samples were removed. To verify genus and/or species assignments, representative sequences of each ASV were compared against those in the NCBI database using the BLAST tool^5^.

### 2.4 Physicochemical analyses

The pH of milk, curd, and cheese samples was measured in triplicate using a pH meter with penetration probe (Crison Instruments, Barcelona, Spain). The acidity (% lactic acid) of the samples was determined in triplicate according to AOAC guidelines (AOAC, 2023). Fat content in milk, curd and cheese was determined in duplicate using a butyrometer, following the methods of van Gulik (ISO 3433; IDF 222, 2008). Water activity (a_w_) was measured in triplicate using an AquaLab apparatus (Decagon Devices Inc., Pullman, WA). Total solids were determined in accordance with the International Standard (IDF 4, 2004).

Total protein (TP) and other nitrogen fractions were determined following standard IDF procedures using the Kjeldahl method (ISO 8968; IDF 20-1, 2014), employing a Kjeldahl-therm KT 20S digestion apparatus (Gerhardt GmbH, Bonn, Germany) and a Vapodest 50 titramatic distillation system (Gerhardt). TP and non-casein nitrogen (NCN) contents were calculated as 6.38 times the total nitrogen content (the standard conversion coefficient for dairy proteins). Non-protein nitrogen (NPN) was calculated using a conversion factor of 6.29. For the determination of NCN, 2 g of cheese were weighed to three decimal places and placed in 100 mL Erlenmeyer flasks. For milk, 6 mL were poured and the weight recorded. A total of 15 mL of distilled water was added, and the samples homogenized in the Stomacher. Each sample was then treated with 5 mL of 10% acetic acid and 5 mL of 10% sodium acetate (Panreac, Madrid, Spain), adjusting the volume to 100 mL with Milli-Q water. The pH was adjusted to 4.6 (the isoelectric point of caseins) using acetic acid (Sigma) and sodium acetate (Panreac). The samples were then heated in a water bath at 45 °C for 45 min and filtered through a 2 μm filter (VWR). To determine NPN, 5 g of cheese and 15 mL of milk were weighed to three decimal places and mixed in the Stomacher with 60 mL of 20% trichloroacetic acid (VWR). The volume was then adjusted to 100 mL with Milli-Q water. The samples were then filtered using 2 μm filters (VWR).

### 2.5 Organic acids and volatile compounds (VOC)

Organic acids and sugars were extracted and determined by HPLC following the method of Alegría et al. (2016), with minor modifications. Briefly, 1 g of cheese was homogenized with 9 mL of 4.25 mM H₂SO₄ using a D100 hand-held homogenizer (Benchmark, Sayreville, NJ, USA). Compounds were separated using an ICSep ICE-ION-300 ion-exchange column (ThermoFisher, Waltham, MA, USA), employing an 8.5 mM H_2_SO_4_ aqueous mobile phase, an operating temperature of 65 °C, and a flow rate of 0.4 mL min^−1^. Organic acids were identified using a 996 Photodiode Array Detector (Waters, Milford, MA, USA) at 210 nm, and sugars using a Waters model 410 differential refractometer at 280 nm. Quantification was performed using calibration curves prepared with commercial standards.

Volatile organic compounds were determined via extraction with methyl tert-butyl ether (MTBE) and targeted analysis by gas chromatography/mass spectrometry (GC/MS). For this, 200 mg of curd and cheese samples were mixed with 1 mL MTBE. The mixtures were shaken for 30 min 1,000 rpm. These samples were then centrifuged, the supernatants collected, and 100 μL mixed with an internal standard and ultrapure water in a GC vial. The vials were vortexed for 2 min and subsequently centrifuged for 2 min. Finally, the organic phase was injected for analysis into a 5975C GC device coupled to a 7890A quadrupole MS (Agilent Technologies, Santa Clara, CA, USA) using a column with optimal selectivity for low to mid polarity compounds (e.g., alcohols, amines, esters, and aromatic hydrocarbons).

A further untargeted analysis of VOC was also performed in duplicate following solid-phase microextraction gas-chromatography (SPME-GC) as reported by Walsh et al. (2020). Briefly, 4 g of finely grated cheese samples were placed in 20 mL screw-capped solid-phase microextraction (SPME) vials (Agilent), which were then sealed with a PTFE/silicone liner septum and equilibrated at 40 °C for 10 min with pulsed agitation for 5 s at 500 rpm using a PAL RSI 120 device (CTC Analytics, Zwingen, Switzerland). VOC were absorbed onto an ARR11-DVB-120/20 DVB/PDMS fiber (CTC Technologies) exposed to the headspace above the samples for 20 min at a depth of 40 mm and at 60 °C. Eluted compounds were identified based on their retention times and by comparison of their mass spectra in the Wiley Mass Spectral Database (Wiley and Sons, NY, USA), with a match score set at >700. Quantification was performed using a GC flame ionization detector (FID) (HP5890 series II plus) (Agilent).

### 2.6 Statistical modeling

Principal component analysis (PCA) was conducted to explore clustering patterns among the Casín cheese samples based on their physicochemical variables, sugars, organic acids, and VOC profiles. Prior to multivariate analysis, the data were pre-processed using UV-scaling (mean-centering and unit variance scaling) to correct for differences in scale among variables. This was performed externally using Python v3.11 and the scikit-learn library (v1. X); the resulting dataset was imported into SIMCA 14.1 (Sartorius Stedim Data Analytics, Umeå, Sweden) for principal component modeling. The PCA model was constructed from the full matrix of UV-scaled data. Principal components were selected based on explained variance (R^2^X) and predictive ability (*Q*^2^), both assessed via cross-validation. Model quality was evaluated using Hotelling’s *T*^2^ and DModX statistics to detect potential outliers. Samples were classified according to their dairy of origin (A, B, or C) using an additional categorical variable and visualized in the scores plot by color-coding. To identify the most influential compounds contributing to sample discrimination, a Loadings Scatter Plot was generated.

Correlation analyses were conducted on an integrated dataset comprising physicochemical variables, VOC concentrations, and microbial community composition. The microbial dataset covered both eukaryotic and prokaryotic taxa. It was pre-processed by filtering to retain only taxa with a mean relative abundance exceeding 0.5% in at least one sample. This filtering step was implemented to emphasize dominant community members and reduce noise from rare taxa. Employing Python with the Pandas module, Pearson correlation coefficients were determined (Pearson, 1896) to detect linear relationships between variables; Spearman rank correlation coefficients (Spearman, 1904) were calculated to capture monotonic relationships, including those that may be non-linear. Correlation coefficients were computed pairwise across microbial taxa and chemical analytes. The resulting correlation matrices were visualized as heatmaps using the Seaborn library in Python. These heatmaps employed color gradients to represent the magnitude and direction of correlations, facilitating the identification of positive and negative associations.

## 3 Results

### 3.1 Basic microbial counts

The trends for most microbial populations over manufacturing and ripening were quite similar for all three batches (Table 1). The highest numbers of majority microbial populations were obtained at day 3 post manufacture, while the highest numbers of subdominant populations were usually obtained on day 7. Total viable mesophilic counts in PCMA reached maximum values of around 9.0 log_10_ colony forming units (CFU) g^−1^ of cheese in batches A and B, and about a half a log lower in batch C. These counts agreed well with those of the LAB populations of lactococci/streptococci on GM17A and *Lactobacillaceae* in MRSA. Subdominant populations, such as enterococci and micrococci/staphylococci, reached maximum cell densities (up to 6.0 CFU g^−1^) three log10 units lower than those of typical LAB populations. *Enterobacteriaceae* and coliforms also reached counts similar to those of enterococci and staphylococci/micrococci, but *Enterobacteriaceae* fell below the limit of detection (<2.0 CFU g^−1^) at day 15 in batches A and C, and at day 60 and in batch B. Yeasts and molds were present in milk at approximately 4.0 CFU mL^−1^, and reached maximum cell densities of >7.00 CFU g^−1^ between day 15 and 60 depending on the batch. Interestingly, all microbial populations fell abruptly (below the limit of detection for all microbial groups except LAB) at day 15 in batch C. This reduction coincided with a mechanical kneading performed by the manufacturer using a meat chopper at around day 10; while producers A and B used a roller kneading machine.

**Table 1:** Viable counts (log_10_ CFU/g) of presumptive microbial groups in three batches of Casín cheese throughout manufacturing and ripening.

Presumptive microbial group (counting medium)	Cheese batch																				
	A							B							C						
	M^a^	C	3 d	7 d	15 d	30 d	60 d	M	C	3 d	7 d	15 d	30 d	60 d	M	C	3 d	7 d	15 d	30 d	60 d
Total viable mesophilic counts (PCAM)	5.75	6.05	8.87	9.03	8.48	8.07	7.30	5.13	5.74	8.99	9.12	8.08	8.13	6.56	5.26	7.91	8.51	8.30	5.04	5.31	3.93
Lactococci/streptococci (GM17A)	5.88	6.86	9.12	9.10	8.57	8.05	7.08	5.32	7.81	9.18	9.18	7.91	8.32	6.95	4.69	7.63	8.40	8.26	4.93	5.08	3.74
*Lactobacillaceae* (MRSA)	5.40	6.43	8.81	8.81	8.35	7.72	7.22	4.62	8.26	8.96	8.19	8.14	8.00	6.57	4.60	6.96	8.03	8.09	4.15	5.02	3.62
Enterococci (SBA)	3.80	4.13	5.73	6.32	5.93	5.93	5.93	3.99	6.23	6.80	7.26	6.30	5.97	5.43	3.85	6.36	4.34	4.08	<2^b^	<2	2.48
Micrococci/staphylococci (BPA)^c^	4.27	5.15	4.94	4.74	4.70	4.95	4.66	4.22	5.47	4.64	6.61	3.36	3.81	3.57	4.39	4.44	5.75	5.97	<2	<2	2.60
*Enterobacteriaceae* (VRBGA)	4.04	4.73	5.42	5.18	<2	<2	<2	4.85	3.84	4.38	5.70	3.44	3.88	<2	3.33	3.87	4.85	6.02	<2	<2	<2
Coliforms (VRBLA)	4.05	4.92	5.39	<2	<2	<2	<2	5.05	4.91	4.19	5.81	3.85	3.08	<2	3.50	3.44	4.66	5.00	<2	<2	<2
Yeasts and molds (YGCA)	4.49	4.79	6.38	6.48	6.66	6.79	7.77	4.33	4.56	6.40	7.15	7.41	7.13	5.07	3.93	3.72	5.21	7.17	<2	4.99	2.90

^a^Samples of milk (M), curd (C) and cheeses of 3, 5, 7, 15, 30, and 60-day after manufacture. ^b^Counts lower than the limit of detection (10^2^ CFU/g or mL). ^c^Colonies in Baird-Parker agar (BPA) surrounded by a clear zone resembling *Staphylococcus aureus* were never recorded.

### 3.2 Metataxonomic profiling

**Figure 1:**
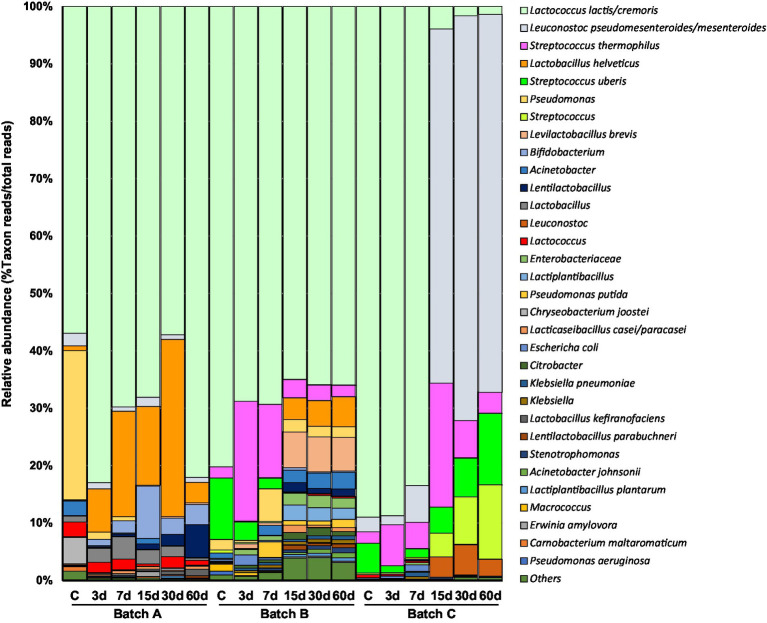
Distribution and relative abundance of bacterial taxa during manufacturing and ripening (samples of curd and 3-, 7-, 15-, 30-, and 60-day old cheeses) of three independent batches of Casín cheese, as identified by 16S rDNA amplification, sequencing, and sequence analysis. The lowest taxonomic rank reached by identification of the reads (which is different for the different phylotypes), is indicated. Only taxa showing a relative abundance >0.5% are depicted.

Raw 16S rDNA paired-end reads from prokaryotes were demultiplexed and merged, which resulted in more than 60,000 sequences per sample. Analysis of the sequences revealed 740 amplicon sequence variants (ASVs) with a mean length of 413 bp. The rarefaction curves of the identified ASVs showed that the sequence coverage reached a plateau in all samples (Supplementary Figure S2A). ASVs were then assigned to 215 bacterial phylotypes (species-, genus-, or family-like taxa, depending on the phylotype), including bacteria from 8 phyla, 80 families, and 116 genera (Supplementary Table S1A). Reads of unidentified taxa at the family and genus levels were also recorded. Each phylotype was represented by a range of 1–11 ASVs. Thirty-two out of the 215 phylotypes (4–18 phylotypes per sample) showed a relative abundance of >0.5% in at least one sample and represented a coverage of 96.1–99.9% of the reads per sample. Thirteen out of the 32 phylotypes were tentatively identified at the species level, 18 at the genus level, and one at the family level only (Figure 1). Overall, 22 bacterial phylotypes were present in at least 50% of the samples, ranging in relative abundance of the reads from 95.51 to 99.89%. Reads of *Lactococcus lactis*/*L. cremoris* (relative abundance 1.40–88.75%; mean 61.16%) were found in all three batches and at all sampling points. These species were declared as starter components by all three producers. *Streptococcus* spp. reads were also abundant in batches B and C (abundance 1.99–30.24%; mean 16.11%). In contrast, *S. thermophilus* was used as a starter by producers A and B. Reads of *Leuconostoc* reflected majority bacterial populations from day 15 onwards in batch C. Surprisingly, *Leuconostoc* species were also among the starter components used by producer A. Several lactobacilli (*Lactobacillus helveticus*, *Levilactobacillus brevis*, and *Lacticaseibacillus casei/L. paracasei*) were found as subdominant populations in two out of the three batches. Large and moderate relative amounts of *L. helveticus* and *L. brevis* reads, respectively, were scored for batch A and B, suggesting strains of these species might also have been found among the starter components. *Bifidobacterium* reads were found in all three batches, being particularly numerous in batch A (relative abundance >9% at day 15).

**Figure 2:**
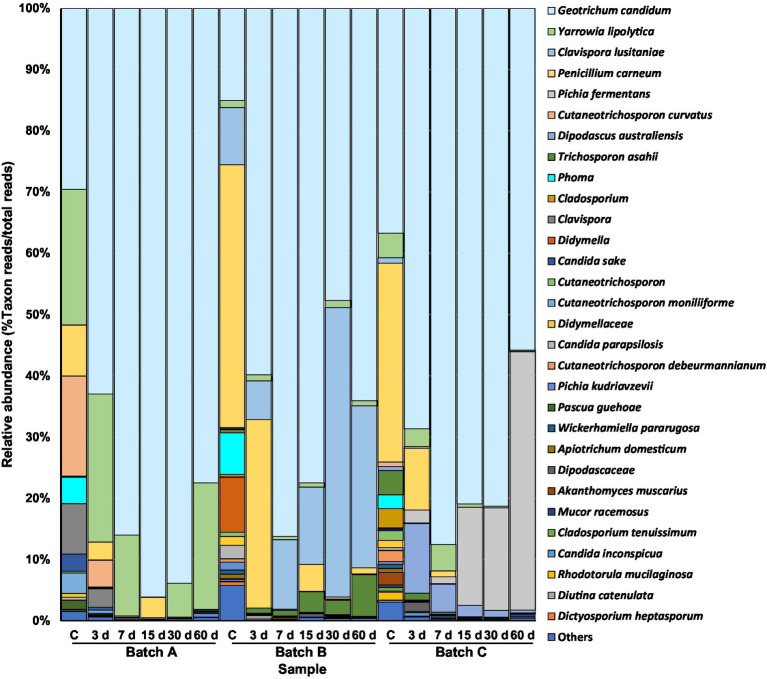
Distribution and relative abundance of fungal taxa during manufacturing and ripening (samples of curd and 3-, 7-, 15-, 30-, and 60-day old cheeses) of three independent batches of Casín cheese, as identified by ITS amplification, sequencing, and sequence analysis. The lowest taxonomic rank reached by identification of the reads (which is different for the different phylotypes), is indicated. Only taxa showing a relative abundance >0.5% are depicted.

For the eukaryotic communities, the number of raw reads per sample was comparable to that obtained for the 16S rDNA datasets. However, the length of the ITS amplicons was greater than the sequencing capability of the technique, and the forward and reverse sequences could not be merged. Since the forward reads showed higher overall quality, these were taken for taxonomic identification. In total, 459 ASVs of the ITS sequences were identified. As for bacteria, the rarefaction curves of eukaryotic ASVs suggested a good coverage for all samples (Supplementary Figure S2B). These were assigned to 204 different fungal phylotypes, including fungi from four phyla, 79 families, and 113 genera (Supplementary Table S1B). A few plant-derived reads and others from unidentified fungal taxa at the family and genus levels were also detected. Phylotypes were composed of 1–41 different ASVs, with the *Geotrichum candidum* phylotype having the greatest number. Thirty out of the 204 phylotypes (with a range of 2–19 taxa per sample) showed a relative abundance of >0.5% (Figure 2). Of the 30 phylotypes, 23 were tentatively identified at the species level, five at the genus level, and two at the family level (Figure 2). Nine eukaryotic phylotypes were observed in at least half of the cheese samples tested, with a relative abundance of 60.8–99.9%. Of these, *G. candidum* and *Yarrowia lipolytica* were present in all cheeses (relative abundance of 16.2–99.4%). The former yeast was detected as the majority relative fungal species in all three Casín batches and at all sampling points (Figure 2). At the subdominant level, distinct yeast species were found in the different batches, with *Y. lipolytica* in batch A, *Clavispora lusitaniae* in batch B, and *Pichia fermentans* in batch C. Reads of subdominant species were found at low relative numbers in curd, increasing over ripening. The filamentous mold *Penicillium carneum* was abundant in curd and in 3-day-old cheese samples from all three batches. However, it was not found in 30- and 60-day-old cheeses, suggesting it did not progress into the cheese matrix.

Only a small number of reads matching genera and species of opportunistic and pathogenic bacteria (e.g., *Escherichia coli*, *Staphylococcus aureus*, *Klebsiella* spp.) were identified. Among these, only small numbers of *E. coli* reads were seen at day 60 in one batch. Further, no reads of *Listeria* species were ever detected in any of the samples (Supplementary Table S1A). Finally, only a few reads of clinically relevant yeast species, such as *Candida sake* and *Pichia kudriavzevii*, were scored at some sampling points. It should be noted that batch B had high relative numbers of reads for *Clavispora lusitaniae –*an emergent pathogenic yeast– throughout manufacturing and ripening.

### 3.3 Basic chemical variables

Table 2 shows the values obtained, during both manufacturing and ripening, for some basic chemical variables. As expected, most values changed over these processes. The lowest pH was obtained for cheese sample on day 3 and 7 for batch A and B, respectively, and cheese samples after kneading for batch C. Most other biochemical variables (acidity, a_w_, dry matter, fat in dry matter, total protein, NCN, and NPN) reached their highest values by the end of ripening (day 60). Note the approximate 10-fold increase in the values for the NCN and NPN fractions from the curd stage to the 60-day-old cheese stage in all batches. The differences scored between batches with respect to some variables, particularly acidity, dry matter, and fat in dry matter, are usual for small scale, artisan dairy products. In contrast, comparable values were recorded for pH, a_w_, total protein, NCN, and NPN in all three batches. These results suggest that the biochemical framework of Casín was maintained in cheeses made by all three producers throughout manufacturing and ripening.

**Table 2:** Basic physico-chemical parameters in three batches of Casín cheese along manufacturing and ripening.

Parameter	Cheese batch																	
	A						B						C					
	C^a^	3 d	7 d	15 d	30 d	60 d	C	3 d	7 d	15 d	30 d	60 d	C	3 d	7 d	**15 d**	**30 d**	**60 d**
pH	6.30 ± 0.04	4.71 ± 0.06	4.75 ± 0.02	5.25 ± 0.02	5.24 ± 0.09	5.30 ± 0.02	6.00 ± 0.4	6.09 ± 0.06	4.98 ± 0.08	5.19 ± 0.07	5.26 ± 0.04	4.98 ± 0.01	4.71 ± 0.01	4.73 ± 0.08	4.79 ± 0.08	4.64 ± 0.05	4.70 ± 0.02	4.67 ± 0.03
a_w_^b^	0.991	0.986	0.985	0.964	0.930	0.872	0.993	0.987	0.966	0.930	0.877	0.875	0.996	0.992	0.987	0.923	0.901	0.885
Titratable acidity (g 100 g^−1^)	0.17 ± 0.03	0.95 ± 0.05	1.23 ± 0.06	1.00 ± 0.06	1.39 ± 0.03	1.28 ± 0.06	0.24 ± 0.05	0.23 ± 0.03	0.98 ± 0.06	1.24 ± 0.05	1.28 ± 0.05	1.72 ± 0.13	0.89 ± 0.03	0.91 ± 0.03	1.09 ± 0.08	2.17 ± 0.03	2.29 ± 0.05	2.88 ± 0.10
Dry matter (%)	46.03	55.52	65.26	65.62	72.55	76.84	38.78	41.20	58.76	64.48	71.35	72.06	52.57	54.37	58.12	61.11	64.66	65.74
Fat in dry matter (%)	44.48 ± 0.4	44.44 ± 0.4	49.53 ± 0.0	49.28 ± 0.0	52.38 ± 0.4	76.85 ± 0.4	61.29 ± 0.0	50.63 ± 0.4	51.18 ± 0.4	51.5 ± 0.0	53.43 ± 0.4	72.06 ± 0.4	48.28 ± 0.	49.46 ± 0.4	53.59 ± 0.4	54.52 ± 0.4	56.66 ± 0.4	65.74 ± 0.4
Total protein (%)	12.44 ± 0.6	15.50 ± 0.8	19.56 ± 0.2	21.04 ± 0.1	23.16 ± 0.7	30.63 ± 3.1	11.22 ± 1.1	14.0 ± 0.7	16.54 ± 0.2	22.43 ± 1.0	26.13 ± 2.4	28.16 ± 1.8	14.16 ± 0.1	17.8 ± 1.9	18.19 ± 0.7	22.56 ± 0.8	23.29 ± 0.5	27.82 ± 1.7
Non-casein nitrogen (%)	0.53 ± 0.04	0.73 ± 0.02	1.54 ± 0.03	2.70 ± 0.05	5.58 ± 0.12	6.70 ± 0.23	0.60 ± 0.01	0.90 ± 0.02	1.33 ± 0.14	4.10 ± 0.10	4.66 ± 0.12	7.19 ± 0.26	0.67 ± 0.03	1.05 ± 0.04	1.80 ± 0.01	5.15 ± 0.20	5.42 ± 0.26	7.58 ± 0.11
Non-protein nitrogen (%)	0.20 ± 0.00	0.46 ± 0.00	0.91 ± 0.04	1.91 ± 0.08	4.2 ± 0.10	4.37 ± 0.08	0.24 ± 0.03	0.45 ± 0.1	0.82 ± 0.14	3.07 ± 0.03	3.4 ± 0.05	4.1 ± 0.08	0.24 ± 0.01	0.37 ± 0.04	0.85 ± 0.01	3.17 ± 0.23	3.77 ± 0.05	5.03 ± 0.23

^a^C, curd samples, and samples of 3, 7, 15, 30, and 60-old day cheeses. ^b^Average ± standard deviation (SD) results; SD 0.0, deviation lower than 0.01.

### 3.4 Analysis of cheese metabolites

Organic acids, sugars and other chemical compounds increased or decreased over manufacturing and ripening in all three batches (Table 3). Lactic, butyric, and acetic acids were the majority organic acids in the ripened cheeses. Lactic and butyric acids continued to increase up to day 60, reaching concentrations above 1 g per 100 g of cheese. Acetic acid also increased in all batches during ripening, but in two out of the three the highest concentration was obtained in 30-day-old cheeses. The citric acid from milk retained in curd was consumed during ripening at variable rates in the different cheeses, in a manner perhaps linked to the microbial starter components. Residual amounts of lactose were still found at day 60 in the ripened cheeses at an average concentration of 294.86 mg 100 g^−1^. Small amounts of ethanol were synthesized during ripening in all batches. Galactose was detected only in batch C 60-day-old cheese.

**Table 3:** Content of organic acids, sugars, and other compounds (mg 100 g^−1^) in three batches of Casín cheese during manufacturing and ripening.

Cheese batch	Sample^a^	Organic acid/compound												
		Acetic^b^	Butyric	Lactic	Orotic	Pyruvic	Uric	Oxalic	Citric	Hippuric	Lactose	Glucose	Galactose	Ethanol
A	M	4.6 ± 2.5	3.4 ± 0.5	24.1 ± 0.1	7.1 ± 0.1	1.0 ± 0.1	1.0 ± 0.1	0.9 ± 0.0	165.8 ± 2.4	4.1 ± 0.3	4851.5 ± 82.1	2.8 ± 0.1	11.7 ± 0.1	-
	C	4.0 ± 0.7	18.2 ± 6.1	7.2 ± 2.0	2.7 ± 0.7	0.2 ± 0.1	0.1 ± 0.1	0.5 ± 0.0	95.1 ± 20.1	1.5 ± 0.6	1792.9 ± 441.2	7.6 ± 2.0	6.5 ± 1.8	-
	3 d	74.5 ± 3.9	18.9 ± 0.6	1198.0 ± 60.5	1.2 ± 0.1	4.8 ± 0.5	–	0.3 ± 0.0	1.8 ± 0.8	–	504.1 ± 41.1	37.5 ± 0.2	46.4 ± 0.8	8.1 ± 0.7
	7 d	64.5 ± 10.0	22.8 ± 2.2	1370.9 ± 107.6	0.8 ± 0.1	3.1 ± 0.3	–	0.7 ± 0.1		–	223.1 ± 7.0	–	1.1 ± 0.1	9.8 ± 0.5
	15 d	82.5 ± 1.1	42.4 ± 0.0	1052.4 ± 13.8	0.9 ± 0.0	2.7 ± 0.2	–	0.3 ± 0.0	–	–	225.9 ± 3.7	–	–	12.0 ± 1.1
	30 d	89.2 ± 3.6	299.2 ± 2.0	1208.4 ± 102.3	0.9 ± 0.0	0.1 ± 0.1	2.0 ± 0.2	0.4 ± 0.0	–	–	275.7 ± 14.5	–	–	9.4 ± 0.0
	60 d	53.9 ± 14.7	393.0 ± 191.7	1213.4 ± 512.8	0.5 ± 0.2	0.9 ± 0.6	0.4 ± 0.3	0.8 ± 0.2	–	–	273.5 ± 36.6	–	–	8.3 ± 0.6
B	M	6.2 ± 0.1	–	–	9.3 ± 0.8	0.3 ± 0.0	0.5 ± 0.0	0.8 ± 0.1	189.0 ± 8.5	1.4 ± 0.1	5257.0 ± 503.2	10.3 ± 0.3	10.0 ± 0.5	–
	C	5.5 ± 1.1	73.5 ± 1.3	13.0 ± 0.0	3.5 ± 0.0	0.3 ± 0.3	–	0.3 ± 0.0	102.6 ± 1.8	0.4 ± 0.0	1956.8 ± 3.5	2.0 ± 0.5	5.7 ± 0.3	5.9 ± 0.3
	3 d	34.6 ± 1.9	4.5 ± 0.3	1038.0 ± 1.7	0.9 ± 0.0	7.5 ± 0.5	–	0.3 ± 0.0	56.3 ± 3.1	–	261.1 ± 1.4	0.5 ± 0.3	99.4 ± 2.9	9.6 ± 0.0.3
	7 d	53.8 ± 14.54	30.7 ± 2.2	902.1 ± 122.6	0.6 ± 0.1	19.0 ± 1.4	0.2 ± 0.1	0.7 ± 0.0	24.5 ± 0.5	–	211.8 ± 5.4	3.3 ± 0.0.2	–	9.7 ± 0.8
	15 d	68.7 ± 1.8	146.8 ± 1.0	774.8 ± 14.2	0.5 ± 0.0	0.4 ± 0.1	–	0.8 ± 0.0	3.3 ± 0.5	–	224.0 ± 7.3	7.3 ± 1.8	–	6.6 ± 1.0
	30 d	103.6 ± 53.7	276.1 ± 150.7	1139.1 ± 607.6	0.7 ± 0.3	1.6 ± 1.0	1.8 ± 1.0	0.7 ± 0.3	2.3 ± 1.0	–	279.9 ± 65.8	2.4 ± 0.1	–	11.1 ± 1.5
	60 d	53.4 ± 7.2	231.0 ± 31.2	770.9 ± 74.1	0.5 ± 0.1	0.1 ± 0.1	0.1 ± 0.0	0.7 ± 0.1	1.9 ± 0.3	–	219.6 ± 10.9	3.4 ± 4.0	–	5.3 ± 1.8
C	M	4.8 ± 2.8	–	–	8.5 ± 0.4	0.5 ± 0.1	0.3 ± 0.0	0.7 ± 0.0	182.6 ± 9.5	0.7 ± 0.0	4902.8 ± 186.6	10.1 ± 0.2	11.5 ± 0.1	–
	C	9.7 ± 0.6	24.4 ± 1.8	71.8 ± 1.5	3.0 ± 0.1	0.1 ± 0.0	0.6 ± 0.1	0.2 ± 0.0	94.4 ± 5.4	–	1713.0 ± 26.8	7.1 ± 0.0	14.5 ± 0.5	9.1 ± 0.9
	3 d	58.4 ± 3.7	8.3 ± 1.7	1090.3 ± 147.9	0.3 ± 0.1	3.2 ± 0.0	–	0.3 ± 0.0	3.0 ± 4.2	–	578.5 ± 67.2	1.4 ± 0.1	69.1 ± 12.1	8.8 ± 0.3
	7 d	35.4 ± 9.2	42.2 ± 16.8	1333.5 ± 413.4	0.3 ± 0.2	2.7 ± 1.2	–	0.9 ± 0.1	8.2 ± 11.5	–	484.6 ± 240.5	15.3 ± 15.1	38.0 ± 6.8	8.6 ± 0.1
	15 d	42.2 ± 27.5	326.4 ± 130.7	1090.4 ± 426.4	0.2 ± 0.0	0.7 ± 0.6	0.2 ± 0.3	0.8 ± 0.4	–	–	287.3 ± 96.6	3.1 ± 1.7	26.9 ± 11.5	6.6 ± 0.2
	30 d	44.9 ± 0.5	500.1 ± 19.0	1432.6 ± 70.1	0.2 ± 0.1	–	2.0 ± 0.0	0.9 ± 0.0	–	–	349.2 ± 12.3	3.2 ± 0.1	35.7 ± 1.5	16.3 ± 0.1
	60 d	53.7 ± 4.5	578.8 ± 19.8	1645.6 ± 92.8	0.4 ± 0.0	–	2.0 ± 0.3	1.2 ± 0.0	–	–	391.5 ± 35.7	4.6 ± 0.2	43.7 ± 2.5	13.7 ± 4.1

^a^M, milk samples; C, curd samples, and samples of 3, 7, 15, 30, and 60-day old cheeses. ^b^The concentration of acetic acid is estimated, as this compound co-elutes with uric acid. –, not detected.

To fully characterize the cheese volatile fraction, targeted and untargeted VOC analyses were performed. Twenty-four VOC were identified by the targeted method after extraction with MTBE (Table 4). Thirteen out of these 24 VOC were detected in most samples. Acetoin, ethyl decanoate, ethyl octanoate, ethyl butyrate, and geraniol were the majority compounds. Some of the compounds increased during ripening, while the concentration of some others decreased or disappeared at some point. For example, acetoin reached a maximum between days 3 and 7, depending on the batch, decreasing afterward during ripening. Fatty acid esters increased over ripening, with the highest values found in cheese at day 60. In addition, 11 compounds were detected in only a few samples and at levels close to the limit of detection or the limit of quantification. Although differences between samples and batches were noted, no VOC profiles that discriminated between the different batches were detected.

**Table 4:** Targeted volatile organic compounds (VOC) as extracted with methyl tert-butyl ether (MTBE) in three batches of Casín cheese during manufacturing and ripening.

Compound^a^	Cheese batch																				
	A							B							C						
	Milk	Curd	3 d	7 d	15 d	30 d	60 d	Milk	Curd	3 d	7 d	15 d	30 d	60 d	Milk	Curd	3 d	7 d	15 d	30 d	**60 d**
Acetoin	100	–	216	54	71	45	51	–	76	313	1,131	162	116	176	27	55	308	97	52	117	–
Citronellyl acetate	–	37	–	–	48	113	–	54	–	–	–	–	92	52	–	48	–	67	60	–	–
Diacetyl	–	–	–	–	–	–	–	–	–	–	48	–	–	–	–	–	–	–	–	–	–
Ethyl acetate	–	–	–	–	–	–	–	–	–	–	64	–	–	–	–	–	–	–	–	–	–
Ethyl butyrate	–	–	4	2	7	27	35	–	–	3	4	24	22	60	–	–	–	–	23	3	25
Ethyl decanoate	40	24	24	–	31	78	99	31	–	–	90	128	107	130	–	30	31	28	357	262	397
Ethyl hexanoate	7	–	–	–	2	3	9	–	–	–	–	4	2	5	–	–	–	–	13	6	12
Ethyl lactate	–	1	1	–	2	3	2	–	–	–	–	1	5	3	–	3	1	6	–	12	1
Ethyl octanoate	–	–	5	–	12	68	97	–	–	–	27	70	40	72	–	–	–	–	287	181	243
Geraniol	–	–	–	–	170	448	–	164	–	–	–	–	424	127	–	–	–	131	137	–	–
Geranyl acetate	–	–	–	–	–	–	–	–	–	–	–	–	–	–	–	–	–	–	–	–	–
Isoamyl acetate	1	1	–	5	1	2	8	3	–	–	–	10	1	–	–	4	3	3	1	5	2
Isoamyl alcohol	–	–	11	–	49	7	3	–	–	–	11	9	2	16	–	2	4	–	3	–	5
Isobutanol	51	13	16	43	22	15	20	16	–	34	14	17	16	19	–	45	14	43	13	43	–
Isobutyl acetate	–	–	–	–	–	–	–	–	–	–	–	–	–	–	–	1	–	1	–	1	–
Linalool	–	–	–	–	–	–	–	–	–	–	–	–	–	3	–	–	1	–	–	–	1
Myrcene	–	–	–	–	–	–	–	–	–	–	–	–	–	–	–	25	22	22	–	–	–
Nerol	190	21	17	–	–	13	13	22	–	14	–	52	–	12	–	40	–	–	–	136	–
2,3–pentanedione	–	0.7	0.4	–	–	–	2.1	0.4	0.3	–	0.5	0.5	–	0.4	–	–	0.5	–	0.3	0.5	–
2–methyl–1–butanol	9	2	5	–	10	3	6	–	–	1	3	7	3	5	–	1	2	1	2	1	2
2–phenyl ethanol	–	–	7	4	15	8	–	4	–	–	7	9	17	16	–	–	–	–	5	–	5
2–phenyl ethyl acetate	–	–	–	2	–	6	–	–	–	–	3	–	3	–	–	–	–	–	–	–	–
4–ethylguaiacol	–	–	–	–	–	–	–	–	–	–	–	–	–	–	–	–	–	10	–	–	5
4–ethylphenol	–	–	5	–	–	–	–	–	–	–	–	–	–	–	–	–	–	–	–	–	–

^a^Quantified as μmol per gram or mL of sample. –, non-detected or lower than the limit of quantification.

The untargeted VOC analysis by the SPME-GC method detected a larger number of compounds. In total, 94 VOC were identified, including 37 esters, 17 acids, 10 ketones, 9 alcohols, 6 lactones, 5 aldehydes, 4 phenols, and 6 compounds of other chemical groups (Supplementary Table S2). Of these, 10 VOC were found in all curd and cheese samples. In decreasing order of relative importance, hexanoic acid, butanoic acid, octanoic acid and n-decanoic acid were the majority VOC, followed by hexanoic acid ethyl ester, octanoic acid ethyl ester, pentatonic acid, heptanoic acid, acetic acid and nonanoic acid. In general, the concentrations of most VOC increased as ripening progressed, reaching their highest relative abundance in 60-day-old samples. Among the differential VOC between batches, hexanoic acid-propyl ester was identified in batches A and B. Decanoic acid methyl ester was detected in batches B and C. Hexanoic acid propyl ester and dimethyl sulphone were identified only in batch A. 1-Methoxy-3-(2-hydroxyethyl)nonane and hexanoic acid-2-methylpropyl ester were only detected in batch B. Two forms of the compounds 2(3H)-furanone-5-butyldihydro, and decanoic acid-ethyl ester were detected only in batch C. Differences in the relative abundance of many other VOC in curds through to 60-day-old cheeses were also scored.

### 3.5 Relationships between variables

**Figure 3:**
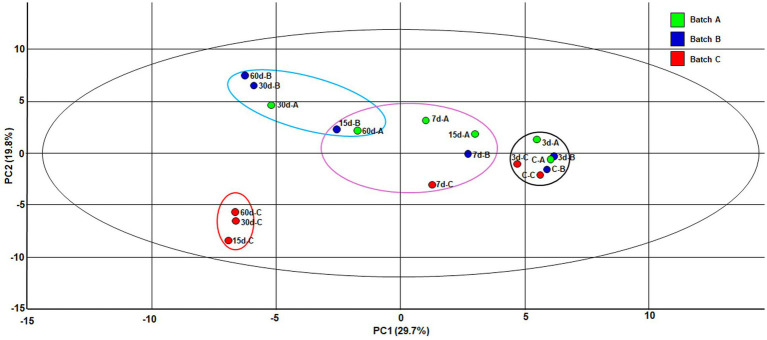
Score plot of the principal component analysis (PCA) of curd (C-A, C-B, C-C) and 3- (3d-A, 3d-B, 3d-C), 7- (7d-A, 7d-B, 7d-C), 15- (15d-A, 15d-B, 15d-C), 30- (30d-A, 30d-B, 30d-C), and 60-day (60d-A, 60d-B, 60d-C) old cheese samples from three independent batches of Casín cheese during manufacturing and ripening.

With no *a priori* hypothesis on causal relationships, a principal component analysis was performed to assess the relationships between producers, ripening time, physicochemical variables and chemical compounds (Figure 3). The two principal components jointly explained 49.5% of the total variance (PC1 29.7% and PC2 19.8%), highlighting the main sources of variability between samples. The score plot revealed a clear separation of samples according to ripening time. This distribution suggests that PC1 primarily reflects the progression of ripening. In contrast, PC2 captures secondary differences between samples, including the batch producer and some physicochemical and chemical variables. Curd samples clustered together irrespective of their producer, as did the majority of 15-, 30-, and 60-day-old cheeses from batch A and B. However, from day 15 onwards, the batch C samples clustered together in the lower left quadrant of the PC1–PC2 plane, far away from all other samples, suggesting they differ in most of the tested variables.

**Figure 4:**
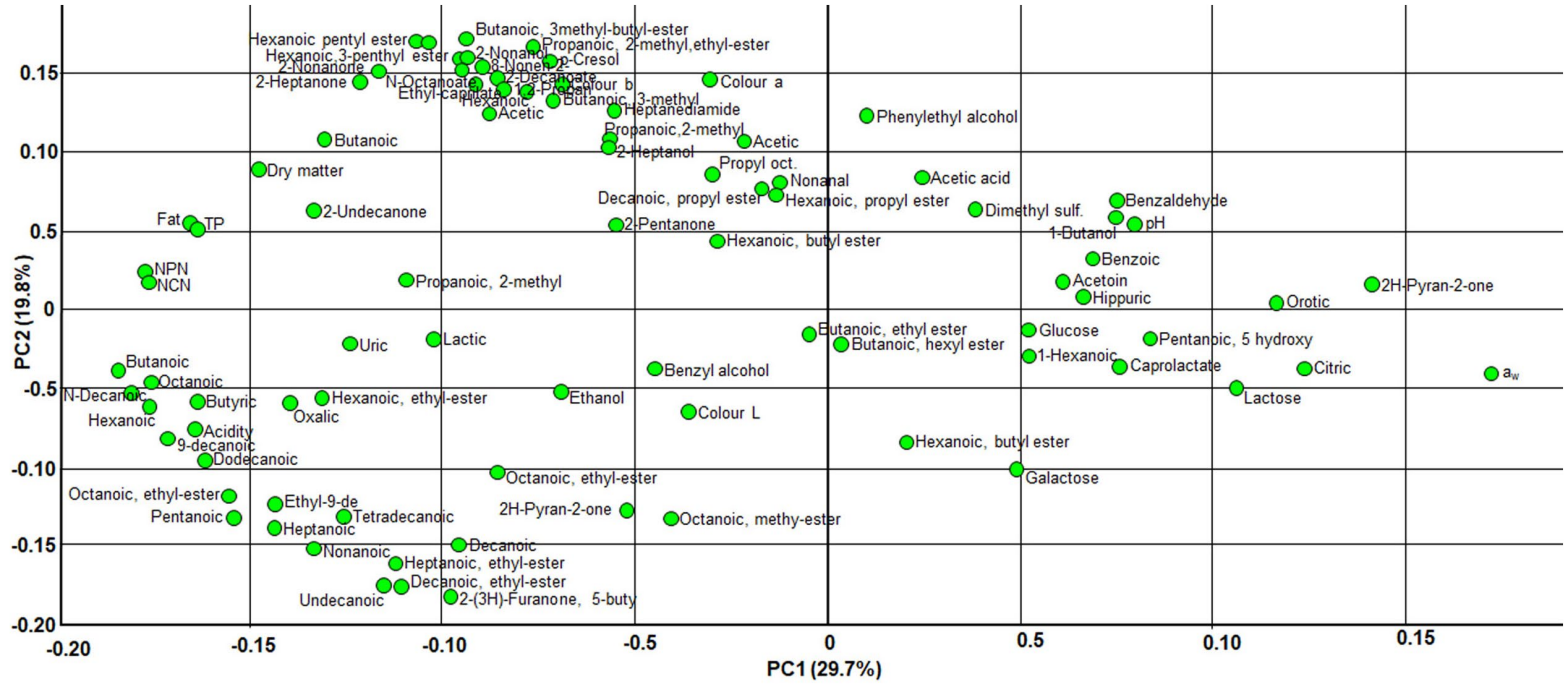
Loading plot showing all physicochemical parameters, and chemical, and volatile compounds from the three batches of Casín cheese used for the PCA.

The loadings plot identified the most discriminant variables responsible for the observed separation of the samples (Figure 4). On PC1, the most negative loadings corresponded to short- and medium-chain fatty acids (butanoic, hexanoic, octanoic, decanoic, and dodecanoic), as well as uric acid, fat, and the NPN fraction –all of which are associated with advanced ripening. In contrast, variables such as a_w,_ lactose, citric acid, galactose, and glucose showed positive loadings, suggesting their association with fresher samples (curds, and 3- and 7-day-old cheese). Although the interpretation of the chemical profiles is complex, since compounds can be formed by chemical processes (e.g., oxidation), have a bacterial origin (e.g., organic acids), or both (with bacterial, rennet or milk enzymes involved), the results indicate a role for lipolysis, proteolysis and carbohydrate utilization in the compositional evolution of Casín cheese. Within the multivariate space defined by the PCA, these biochemical processes contribute significantly to the differentiation of samples over ripening and between the batches made by the different producers.

**Figure 5:**
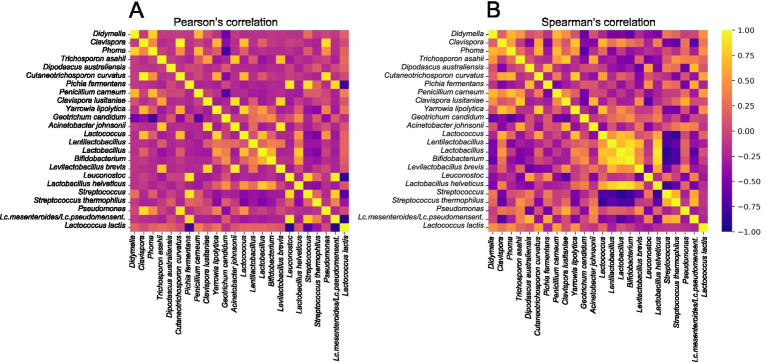
Heat map of Personʼs **(A)** and Spearmanʼs **(B)** co-occurrence and co-exclusion between the 14-majority bacterial and fungal populations along manufacturing and ripening of Casín cheese. Yellow and deep purple colors indicate positive and negative correlations, respectively.

**Figure 6:**
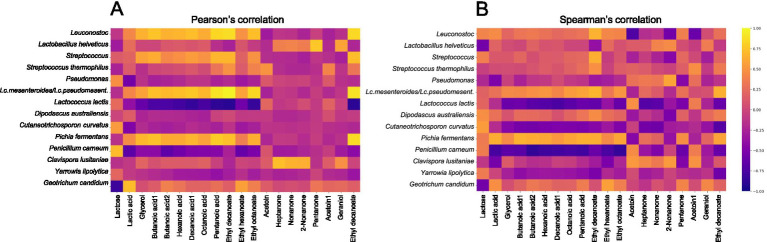
Heat map of non-parametric Personʼs **(A)** and Spearmanʼs **(B)** correlations between 14 majority bacterial and fungal populations and 20 chemical parameters and volatile compounds along manufacturing and ripening of Casín cheese. Yellow and deep purple colors indicate positive and negative correlations, respectively.

Pearson and Spearman correlations between microbes, and between microbes and analytes, were determined (Figures 5, 6). Given the different statistical nature of the correlations (linear versus monotonic), differences in intensity in positive and negative correlations for the same variables were obtained. In general, Pearson correlations were more discriminat than those obtained by the Spearman test. Among the microbial correlations, a strong positive correlation was detected between *Leuconostoc* spp. and *P. fermentans* (Figure 5). *L. lactis* showed a negative correlation with *Leuconostoc* spp., *Streptococcus* spp., and *P. fermentans*. *C. lusitaniae* showed a positive correlation with *Acinetobacter johnsonii* and *Levilactoacillus brevis*, while *G. candidum* showed a strong negative correlation with fungi of the genera *Didymella* and *Phona*, and the species *Penicillium carneum*. Regarding microbial and analytical correlations, a strong positive association was observed between *Leuconostoc* spp. and *P. fermentans*, and the metabolites pentatonic acid, ethyl ester decanoic acid, ethyl decanoate, and ethyl ester octanoic acid (Figure 6). Surprisingly, *L. lactis* correlated negatively with a majority of the compounds, including lactic acid.

## 4 Discussion

The molecular, culture-independent technique used in this work has expanded the spectrum of bacterial and fungal communities present over the manufacturing and ripening of Casín cheese. The metabolites of microbial origin, including organic acids and VOC were also identified and quantified. In addition, multivariate analysis correlated the majority microbial populations among themselves, and with main physicochemical and biochemical profiles of this cheese.

The differences noted in counts of microbial populations between the examined batches may have arisen through differences in the milk used by the manufactures or from minor differences in manufacturing practices (e.g., prematuration or not of the cheese milk, type of starter culture, type and amount of rennet, etc.). In agreement with previous results on a single cheese batch (Alegría et al., 2009), culture-dependent and molecular methods showed LAB to form the majority populations in Casín, from curd through ripened cheeses. Comparably large LAB populations have been reported in many cheeses, particularly in those manufactured with starters, such as Herve (Delcenserie et al., 2014), Robiola di Roccaverano (Biolcati et al., 2022), Serra da Canastra (Kamimura et al., 2020), May Brynza (Pangallo et al., 2014), and others. Starter LAB are deliberately added to milk to control the fermentation and standardize cheese quality (Fox et al., 2017a). Non-starter LAB (NSLAB) are also frequently detected by both culturing and molecular methods in many cheese types (Biolcati et al., 2022; Choi et al., 2020; Decadt et al., 2024; Escobar-Zepeda et al., 2016; Papadimitriou et al., 2022). The NSLAB species detected in the present study belonged to homofermentative or facultative heterofermentative lactobacilli species, including some from the genera *Lactobacillus*, *Lacticaseibacillus*, *Levilactobacillus*, and *Lantiplantibacillus*. These originate from either the starter, the milk, or the manufacturing and ripening environments (Frétin et al., 2022; Settanni and Moschetti, 2010). NSLAB species are thought to contribute mostly to the secondary proteolysis required for full flavor development (Choi et al., 2020; Peralta et al., 2017). Overall, starter LAB and NSLAB contribute to the final texture and flavor in cheese via the utilization of lactose and citrate, and via the catabolic pathways associated with their proteolytic and lipolytic activities (Blaya et al., 2018). It is noteworthy that cultivable *Enterobacteriaceae* and coliforms were no longer detectable in batches A and C from day 15 onward, whereas these groups persisted in batch B up to day 60. Such discrepancies may be attributable to differences in hygienic practices, the native microbiota of the raw milk, or environmental conditions during cheese manufacture and ripening (Chaves-López et al., 2006). Although bacteria belonging to these taxa may influence the development of cheese sensory attributes (Chaves-López et al., 2006; Morales et al., 2004), the potential occurrence of pathogenic species within this family represents a possible public health concern. As regards the eukaryotic populations, and in agreement with our previous study (Alegría et al., 2009), large viable numbers of yeasts and molds in Casín –the majority populations in the ripened cheeses of most batches– were found. Members of the mycobiota may produce mycotoxins, representing a potential hazard to consumers, and are responsible for cheese spoilage, resulting in significant wastes and economic losses (Cenci-Goga et al., 2021). However, yeasts and molds may also play a key role in the formation of flavor, aroma, texture and appearance in many cheeses (Cenci-Goga et al., 2021).

In agreement with other studies on traditional and industrial cheeses using NGS technologies (Alessandria et al., 2017; Ceugniez et al., 2017a; Ceugniez et al., 2017b; Ercolini et al., 2012; Escobar-Zepeda et al., 2016; Lessard et al., 2014; Monnet et al., 2016), a wide diversity of prokaryotic and eukaryotic microorganisms was found by the metataxonomic analysis over the Casín manufacturing and ripening stages. Due to the addition of LAB starters, the diversity of bacteria was similar in all samples, while fungi showed the greatest number of taxa in curd samples. This fungal diversity decreased sharply from day 3 onwards, reaching comparable numbers of taxa to those for bacteria at day 60. Beyond the majority bacterial and fungal species, little was known about the technological, quality and safety significance of the large series of previously undetected prokaryotic and eukaryotic organisms present during manufacturing and ripening. The presence of reads with no match in current databases, some of which could not be assigned even at the family or genus level, suggests that the microbiota of Casín cheese is indeed very complex. The presence of microorganisms without culture representation in several foods, but particularly in fermented dairy products, has recently been stressed (Carlino et al., 2024). It is thought that only phylotypes present in certain amounts (e.g., >0.5 or 1%) contribute to the sensory properties of the cheese. However, organisms present in small amounts could still have a strong influence if they have unique metabolic properties. For example, small relative numbers of *Brevibacterium* and *Pediococcus* have been suggested to influence the flavor profiles of artisanal Irish cheeses (Quigley et al., 2012; Eugster et al., 2019). The importance of other bacteria, such as the gut-associated *Bifidobacterium* or *Ruminococcaceae* detected in this study and others (Alegría et al., 2012; OʼSullivan et al., 2015), remains to be determined.

Among the many fungal species detected by the molecular technique, a few (*G. candidum, K. marxianus, Debaryomyces hansenii, Y. lipolytica,* and *Pichia* spp.) have been shown to be majority populations in cheeses and cheese-associated ecosystems (Anelli et al., 2019; Biolcati et al., 2022; Marín et al., 2015; Pangallo et al., 2014). However, the majority fungal species might be specific for different cheese varieties. As such, *Candida* spp. has been reported as the majority yeast species in Serra da Estrela cheese (Rocha et al., 2021), and *D. hansenii* in Gouda cheese (Decadt et al., 2024). *G. candidum* was the major species present in all samples of the present Casín cheese; particularly after day 3 post-manufacture. Denaturing gradient gel electrophoresis has already shown this yeast to form majority fungal populations in Casín (Alegría et al., 2009). *G. candidum* utilizes lactic acid, contributing to the neutralization of the cheese matrix, favoring the development of acid-susceptible microbial populations (Kamilari et al., 2023). It also releases proteases, lipases and other enzymes that generate taste and aroma compounds or their precursors (Kamilari et al., 2023).

Comparison of the culture- and amplicon-determined microbial populations allowed for estimates of viable but non-culturable (VBNC) species to be made. No viable microorganisms of some bacterial populations could be recovered from 15 and 30 day-old samples from batch C, while no appreciable changes were observed in the metataxonomic profiles. The mechanical chopping of the cheese performed by this producer around day 10 might have caused a concomitant release of free fatty acids (or other substances) with strong antimicrobial activity (Clément et al., 2008). In agreement with this observation, the titratable acidity (Table 2) and the butyric acid concentration in batch C from day 15 onward were substantially higher than those in the other two batches (Table 3), which may have contributed to a reduction in the cultivability of all microbial populations, either through cell death or transition into a VBNC state. Nonetheless, dead cells, and those in a VBNC state, still release proteases, peptidases, lipases, and other hydrolytic enzymes into the cheese matrix, contributing to the development of aroma and taste (Carpino et al., 2017; Fox et al., 2017b). For batch C, it would be interesting to determine whether the taxa present at day 60 stem from survivors of the majority populations at day 7, or belong to treatment-resistant species found in small numbers.

Metabolic profiling by HPLC, plus targeted and untargeted GC-MC, revealed an array of organic acids and VOC contributing to the flavor profile of Casín cheese. Many compounds were present throughout manufacturing and ripening. However, the pivotal ones might be those that are synthesized during ripening, or that increase significantly during this period. Carboxylic acids are reported to be majority flavor compounds in many cheese types (Wang et al., 2021). They also serve as precursors for the synthesis of methyl ketones, alcohols, lactones and esters (Wang et al., 2020). Hexanoic, butanoic, octanoic and n-decanoic acids were the majority VOC in the present cheese –acids mostly formed by lipolysis of the milk fat (Fox et al., 2017b). At high concentrations, carboxylic acids have pungent flavors (Wang et al., 2021), which is consistent with the strong aroma and taste of mature Casín cheeses. Ethyl esters of fatty acids are also known for their important role in the formation of a fruity character in cheese (Wang et al., 2021). In the present study, ethyl decanoate, ethyl octanoate and ethyl butyrate were the majority esters in all samples. The presence in Casín cheese of moderate amounts alcohol, which is required for the synthesis of ethyl esters (Richoux et al., 2008), suggest the formation of these VOC is not limited by the absence of this precursor. Geraniol, one of the compounds detected, is a naturally-occurring monoterpene alcohol with a geranium-like odor. This has also been detected in Cantal cheese (Cornu et al., 2005). It is thought to proceed from milk produced by grass-grazing cows, suggesting the present cheeses were made from such milk.

The associations identified through correlation analyses do not necessarily imply causality unless supported by biochemical or molecular evidence. For instance, the strong positive correlation observed between *Leuconostoc* spp. and *P. fermentans* may be attributed to ethanol production via the heterofermentative metabolism of the bacteria, which supplies a key precursor for ester synthesis by the yeast (Dzialo et al., 2017; Liu et al., 2004). Alternatively, such correlations can serve as a foundation for formulating hypotheses to be tested in future studies. One of the most interesting associations is the negative correlation between *G. candidum* and several mold species. Certainly, the competition between *G. candidum* and *Mucor* species is a very old cheesemaker’s empirical observation (Le Bars-Bailly et al., 1999; Tormo and Barral, 2025).

Although moderate numbers of *Enterobacteriaceae* and coliforms may be counted during the manufacturing and ripening of Casín (Alegría et al., 2009; Table 1), the small relative numbers of reads for these groups agrees well with their susceptibility to lactic acid and the absence of viable cells in all batches at day 60. These results, together with the absence of viable cells and sequencing reads of other pathogens at the end of ripening –such as *S. aureus* and *L. monocytogenes*– suggest that, despite being produced from raw milk, Casín cheese is safe for consumption.

## 5 Conclusion

Metataxonomic profiling of the microbial populations enabled the identification of a wide range of eukaryotic and prokaryotic phylotypes over Casín cheese production and ripening. Previously uncultured bacterial and fungal taxa were also detected. Among the taxa with cultured representatives, only *L. lactis/L. cremoris* and *G. candidum* consistently dominated across all samples and batches. A mixture of lactococci and *G. candidum* strains is proposed as the minimal microbiota-based starter for Casín cheese. *Lc. mesenteroides/Lc. pseudomesenteroides* was shown as the majority population from day 15 onwards in batch C. Other LAB species, such as *L. helveticus* and *S. thermophilus* constituted subdominant populations in certain batches. These are believed to exert the most significant influence on the chemical and sensory profiles of cheese. Metabolic profiling of the organic acids and VOC produced over manufacturing and ripening revealed a wide array of compounds, most of which are presumed to be of microbial origin. The statistical correlations found between the dominant species and the analytical data must be tested experimentally. To this end, mixtures of lactococci and *G. candidum*, either on their own or supplemented with *Leuconostoc* spp., *S. thermophilus* or *L. helveticus* strains, will be tested and evaluated in experimental trials conducted under real Casín cheese manufacturing conditions. This will enable the most effective combination of species for enhancing overall cheese quality and safety to be identified.

## Data Availability

Metataxonomic data have now been deposited in the Sequence Read Archive (SRA) of the NCBI database (http://www.ncbi.nlm.nih.gov, accessed on 25th November 2024) under the accession numbers of Bioproject PRJNA1368803, Biosamples SAMN53372391-SAMN53372411.

## Footnotes

1
https://www.mapa.gob.es/es/alimentacion/temas/calidad-diferenciada/dop-igp/detalle/queso_lacteo/dop_casin

2
http://qiime.org/

3
https://github.com/marcomeola/DAIRYdb

4
https://unite.ut.ee/

5
http://www.ncbi.nlm.nih.gov/blast/

## References

[ref1] Afshari R. Pillidge C. J. Dias D. A. Osborn A. M. Gill H. (2020). Cheesomics: the future pathway to understanding cheese flavour and quality. Crit. Rev. Food Sci. Nutr. 60, 33–47. doi: 10.3390/foods11020188 30285475

[ref2] Alegría A. Alvarez-Martín P. Sacristán N. Fernández E. Delgado S. Mayo B. (2009). Diversity and evolution of the microbial populations during manufacture and ripening of Casín, a traditional Spanish, starter-free cheese made from cowʼs milk. Int. J. Food Microbiol. 136, 44–51. doi: 10.1016/j.ijfoodmicro.2009.09.023, 19822375

[ref3] Alegría A. González P. Delgado S. Flórez A. B. Hernández-Barranco A. Rodríguez A. . (2016). Characterisation of the technological behaviour of mixtures of mesophilic lactic acid bacteria isolated from traditional cheeses made of raw milk without added starters. Int. J. Dairy Technol. 69, 1–13. doi: 10.1111/1471-0307.12253

[ref4] Alegría A. Szczesny P. Mayo B. Bardowski J. Kowalczyk M. (2012). Biodiversity in Oscypek, a traditional polish cheese, determined by culture-dependent and -independent approaches. Appl. Environ. Microbiol. 78, 1890–1898. doi: 10.1128/AEM.06081-11, 22247135 PMC3298175

[ref5] Alessandria V. Ferrocino I. De Filippis F. Fontana M. Rantsiou K. Ercolini D. . (2017). Microbiota of an Italian grana-like cheese during manufacture and ripening, unraveled by 16S rRNA-based approaches. Appl. Environ. Microbiol. 82, 3988–3995. doi: 10.1128/AEM.00999-16 PMC4907185 27107125

[ref6] Anelli P. Haidukowski M. Epifani F. Cimmarusti M. T. Moretti A. Logrieco A. . (2019). Fungal mycobiota and mycotoxin risk for traditional artisan Italian cave cheese. Food Microbiol. 78, 62–72. doi: 10.1016/j.fm.2018.09.014, 30497609

[ref7] AOAC (2023). Official methods of analysis of the association analytical chemists. 22nd Edn. Maryland, USA: Association of Official Analytical Chemists.

[ref8] Biolcati F. Ferrocino I. Bottero M. T. Dalmasso A. (2022). The bacterial and fungal microbiota of "Robiola di Roccaverano" protected designation of origin raw milk cheese. Front. Microbiol. 12:776862. doi: 10.3389/fmicb.2021.776862, 35173686 PMC8841559

[ref9] Blaya J. Barzideh Z. LaPointe G. (2018). Symposium review: interaction of starter cultures and nonstarter lactic acid bacteria in the cheese environment. J. Dairy Sci. 101, 3611–3629. doi: 10.3168/jds.2017-13345, 29274982

[ref11] Bolyen E. Rideout J. R. Dillon M. R. Bokulich N. A. Abnet C. C. Al-Ghalith G. A. . (2019). Reproducible, interactive, scalable and extensible microbiome data science using QIIME 2. Nat. Biotechnol. 37, 852–857. doi: 10.1038/s41587-019-0209-9, 31341288 PMC7015180

[ref12] Callahan B. J. McMurdie P. J. Rosen M. J. Han A. W. Johnson A. J. Holmes S. P. (2016). DADA2: high-resolution sample inference from Illumina amplicon data. Nat. Methods 13, 581–583. doi: 10.1038/nmeth.3869, 27214047 PMC4927377

[ref13] Caporaso J. G. Lauber C. L. Walters W. A. Berg-Lyons D. Huntley J. Fierer N. . (2012). Ultra-high-throughput microbial community analysis on the Illumina HiSeq and MiSeq platforms. ISME J. 6, 1621–1624. doi: 10.1038/ismej.2012.8, 22402401 PMC3400413

[ref14] Carlino N. Blanco-Míguez A. Punčochář M. Mengoni C. Pinto F. Tatti A. . (2024). Unexplored microbial diversity from 2,500 food metagenomes and links with the human microbiome. Cell 187, 5775–5795.e15. doi: 10.1016/j.cell.2024.07.039, 39214080

[ref15] Carpino S. Randazzo C. L. Pino A. Russo N. Rapisarda T. Belvedere G. . (2017). Influence of PDO Ragusano cheese biofilm microbiota on flavour compounds formation. Food Microbiol. 61, 126–135. doi: 10.1016/j.fm.2016.09.006, 27697162

[ref16] Cenci-Goga B. Cruciani D. Crotti S. Karama M. Yıldırım G. Bulut M. . (2021). Diversity of yeasts and moulds in dairy products from Umbria, Central Italy. J. Dairy Res. 88, 217–220. doi: 10.1017/S002202992100042X, 33985601

[ref17] Ceugniez A. Taminiau B. Coucheney F. Jacques P. Delcenserie V. Daube G. . (2017a). Fungal diversity of "Tomme d'Orchies" cheese during the ripening process as revealed by a metagenomic study. Int. J. Food Microbiol. 258, 89–93. doi: 10.1016/j.ijfoodmicro.2017.07.015, 28806689

[ref18] Ceugniez A. Taminiau B. Coucheney F. Jacques P. Delcenserie V. Daube G. . (2017b). Use of a metagenetic approach to monitor the bacterial microbiota of “Tomme d'Orchies” cheese during the ripening process. Int. J. Food Microbiol. 247, 65–69. doi: 10.1016/j.ijfoodmicro.2016.10.034, 27817942

[ref19] Chaves-López C. De Angelis M. Martuscelli M. Serio A. Paparella A. Suzzi G. (2006). Characterization of the *Enterobacteriaceae* isolated from an artisanal Italian ewe's cheese (pecorino Abruzzese). J. Appl. Microbiol. 101, 353–360. doi: 10.1111/j.1365-2672.2006.02941.x, 16882142

[ref20] Chen S. Zhou Y. Chen Y. Gu J. (2018). Fastp: an ultra-fast all-in-one FASTQ preprocessor. Bioinformatics 34, i884–i890. doi: 10.1093/bioinformatics/bty560, 30423086 PMC6129281

[ref1104] Choi J. In Lee S. Rackerby B. Frojen R. Goddik L. Ha S. D. . (2020). Assessment of overall microbial community shift during Cheddar cheese production from raw milk to aging. Appl. Microbiol. Biotechnol. 104, 6249–6260. doi: 10.1007/s00253-020-10651-7 32451588

[ref21] Clément M. Tremblay J. Lange M. Thibodeau J. Belhumeur P. (2008). Purification and identification of bovine cheese whey fatty acids exhibiting in vitro antifungal activity. J. Dairy Sci. 91, 2535–2544. doi: 10.3168/jds.2007-0806, 18565910

[ref22] Cornu A. Kondjoyan N. Martin B. Verdier-Metz I. Pradel P. Berdagué J.-L. . (2005). Terpene profiles in Cantal and saint-Nectaire-type cheese made from raw or pasteurised milk. J. Sci. Food Agric. 85, 2040–2046. doi: 10.1002/jsfa.2214

[ref23] Decadt H. Weckx S. de Vuyst L. (2024). The microbial and metabolite composition of gouda cheese made from pasteurized milk is determined by the processing chain. Int. J. Food Microbiol. 412:110557. doi: 10.1016/j.ijfoodmicro.2024.110557, 38237418

[ref1107] Delcenserie V. Taminiau B. Delhalle L. Nezer C. Doyen P. Crevecoeur S. . (2014). Microbiota characterization of a Belgian protected designation of origin cheese, Herve cheese, using metagenomic analysis. J. Dairy Sci. 97, 6046–6056. doi: 10.3168/jds.2014-8225, 25064656

[ref24] Dzialo M. C. Park R. Steensels J. Lievens B. Verstrepen K. J. (2017). Physiology, ecology and industrial applications of aroma formation in yeast. FEMS Microbiol. Rev. 41, S95–S128. doi: 10.1093/femsre/fux031, 28830094 PMC5916228

[ref25] Ercolini D. De Filippis F. La Storia A. Iacono M. (2012). “Remake” by high-throughput sequencing of the microbiota involved in the production of water buffalo mozzarella cheese. Appl. Environ. Microbiol. 78, 8142–8145. doi: 10.1128/AEM.02218-12, 22941080 PMC3485941

[ref1106] Escobar-Zepeda A. Sanchez-Flores A. Quirasco Baruch M. (2016). Metagenomic analysis of a Mexican ripened cheese reveals a unique complex microbiota. Food Microbiol. 57, 16–27. doi: 10.1016/j.fm.2016.02.004 27052710

[ref26] Eugster E. Fuchsmann P. Schlichtherle-Cerny H. Bütikofer U. Irmler S. (2019). Formation of alanine, α-aminobutyrate, acetate, and 2-butanol during cheese ripening by *Pediococcus acidilactici* FAM18098. Int. Dairy J. 96, 21–28. doi: 10.1016/j.idairyj.2019.04.001

[ref27] Fox P. F. Guinee T. P. Cogan T. M. McSweeney P. L. H. (2017a). “Microbiology of cheese ripening” in Fundamentals of cheese science. eds. Fox P. F. Guinee T. P. Cogan T. M. McSweeney P. L. H. (Boston, MA, USA: Springer), 333–390.

[ref28] Fox P. F. Guinee T. P. Cogan T. M. McSweeney P. L. H. (2017b). “Biochemistry of cheese ripening” in Fundamentals of cheese science. eds. Fox P. F. Guinee T. P. Cogan T. M. McSweeney P. L. H. (Boston, MA, USA: Springer), 391–442.

[ref29] Frétin M. Gérard A. Ferlay A. Martin B. Buchin S. Theil S. . (2022). Integration of multiomic data to characterize the influence of milk fat composition on Cantal-type cheese microbiota. Microorganisms 10:334. doi: 10.3390/microorganisms10020334, 35208788 PMC8879305

[ref30] IDF 20-1. (2014). Milk and milk products -determination of nitrogen content part 1: Kjeldahl principle and crude protein calculation. Brussels.

[ref31] IDF 222. (2008). Cheese – determination of fat content – Van Gulik method. Brussels.

[ref32] IDF 4. (2004). Cheese and processed cheese -determination of the total solids content. Brussels.

[ref33] Jin H. Mo L. Pan L. Hou Q. Li C. Darima I. . (2018). Using PacBio sequencing to investigate the bacterial microbiota of traditional Buryatian cottage cheese and comparison with Italian and Kazakhstan artisanal cheeses. J. Dairy Sci. 101, 6885–6896. doi: 10.3168/jds.2018-14403, 29753477

[ref34] Kamilari E. Stanton C. Reen F. J. Ross R. P. (2023). Uncovering the biotechnological importance of *Geotrichum candidum*. Foods 12:1124. doi: 10.3390/foods12061124, 36981051 PMC10048088

[ref1108] Kamimura B. A. Cabral L. Noronha M. F. Baptista R. C. Nascimento H. M. Sant’Ana S. (2020). Amplicon sequencing reveals the bacterial diversity in milk, dairy premises and Serra da Canastra artisanal cheeses produced by three different farms. Food Microbiol. 89:103453. doi: 10.1016/j.fm.2020.103453 32138999

[ref35] Kõljalg U. Nilsson R. H. Abarenkov K. Tedersoo L. Taylor A. F. Bahram M. . (2013). Towards a unified paradigm for sequence-based identification of fungi. Mol. Ecol. 22, 5271–5277. doi: 10.1111/mec.12481 24112409

[ref36] Le Bars-Bailly S. Bailly J. D. Brugere H. (1999). Mold-related failings in cheesemaking. Rev. Med. Vet. 150, 413–430.

[ref37] Lee J. Seo Y. Ha J. Kim S. Choi Y. Oh H. . (2020). Influence of milk microbiota on *Listeria monocytogenes* survival during cheese ripening. Food Sci. Nutr. 8, 5071–5076. doi: 10.1002/fsn3.1806, 32994967 PMC7500772

[ref38] Lessard M. H. Viel C. Boyle B. St-Gelais D. Labrie S. (2014). Metatranscriptome analysis of fungal strains *Penicillium camemberti* and *Geotrichum candidum* reveal cheese matrix breakdown and potential development of sensory properties of ripened camembert-type cheese. BMC Genomics 15:235. doi: 10.1186/1471-2164-15-235, 24670012 PMC3986886

[ref39] Levante A. Bertani G. Marrella M. Mucchetti G. Bernini V. Lazzi C. . (2023). The microbiota of mozzarella di Bufala Campana PDO cheese: a study across the manufacturing process. Front. Microbiol. 14:1196879. doi: 10.3389/fmicb.2023.1196879, 37649628 PMC10462780

[ref40] Li J. Zheng Y. Xu H. Xi X. Hou Q. Feng S. . (2017). Bacterial microbiota of Kazakhstan cheese revealed by single molecule real time (SMRT) sequencing and its comparison with Belgian, Kalmykian and Italian artisanal cheeses. BMC Microbiol. 17:13. doi: 10.1186/s12866-016-0911-4, 28068902 PMC5223556

[ref41] Liu S.-Q. Holland R. Crow V. L. (2004). Esters and their biosynthesis in fermented dairy products: a review. Int. Dairy J. 14, 923–945. doi: 10.1016/j.idairyj.2004.02.010

[ref42] Marín P. Palmero D. Jurado M. (2015). Occurrence of moulds associated with ovine raw milk and cheeses of the Spanish region of Castilla La Mancha. Int. J. Dairy Technol. 68, 565–572. doi: 10.1111/1471-0307.12208

[ref1105] Martin M. (2011). Cutadapt removes adapter sequences from high-throughput sequencing reads. EMBnet.journal 17, 10–12. doi: 10.14806/ej.17.1.200

[ref43] Martin K. J. Rygiewicz P. T. (2005). Fungal-specific PCR primers developed for analysis of the ITS region of environmental DNA extracts. BMC Microbiol. 5:28. doi: 10.1186/1471-2180-5-28, 15904497 PMC1156903

[ref44] Meola M. Rifa E. Shani N. Delbès C. Berthoud H. Chassard C. (2019). DAIRYdb: a manually curated reference database for improved taxonomy annotation of 16S rRNA gene sequences from dairy products. BMC Genomics 20:560. doi: 10.1186/s12864-019-5914-8, 31286860 PMC6615214

[ref45] Monnet C. Dugat-Bony E. Swennen D. Beckerich J.-M. Irlinger F. Fraud S. . (2016). Investigation of the activity of the microorganisms in a reblochon-style cheese by metatranscriptomic analysis. Front. Microbiol. 7:536. doi: 10.3389/fmicb.2016.00536, 27148224 PMC4837152

[ref46] Morales P. Feliu I. Fernández-García E. Nuñez M. (2004). Volatile compounds produced in cheese by *Enterobacteriaceae* strains of dairy origin. J. Food Prot. 67, 567–573. doi: 10.4315/0362-028x-67.3.567, 15035375

[ref47] Nam J. H. Cho Y. S. Rackerby B. Goddik L. Park S. H. (2021). Shifts of microbiota during cheese production: impact on production and quality. Appl. Microbiol. Biotechnol. 105, 2307–2318. doi: 10.1007/s00253-021-11201-5, 33661344

[ref48] OʼSullivan D. J. Cotter P. D. OʼSullivan O. Giblin L. McSweeney P. L. Sheehan J. J. (2015). Temporal and spatial differences in microbial composition during the manufacture of a continental-type cheese. Appl. Environ. Microbiol. 81, 2525–2533. doi: 10.1128/AEM.04054-14 25636841 PMC4357954

[ref49] Pangallo D. Saková N. Koreňová J. Puškárová A. Kraková L. Valík L. . (2014). Microbial diversity and dynamics during the production of may Bryndza cheese. Int. J. Food Microbiol. 170, 38–43. doi: 10.1016/j.ijfoodmicro.2013.10.015, 24291178

[ref1102] Papadimitriou K. Anastasiou R. Georgalaki M. Bounenni R. Paximadaki A. Charmpi C. . (2022). Comparison of the Microbiome of Artisanal Homemade and Industrial Feta Cheese through Amplicon Sequencing and Shotgun Metagenomics. Microorganisms 10, 1073. doi: 10.3390/microorganisms10051073, 35630516 PMC9146562

[ref50] Pearson K. (1896). Mathematical contributions to the theory of evolution. Part III. Regression, heredity, and panmixia. Philos. Trans. R. Soc. Lond. A 187, 253–318.

[ref51] Peralta G. H. Bergamini C. V. Audero G. Páez R. Wolf I. V. Perotti M. C. . (2017). Spray-dried adjunct cultures of autochthonous non-starter lactic acid bacteria. Int. J. Food Microbiol. 255, 17–24. doi: 10.1016/j.ijfoodmicro.2017.05.014, 28558330

[ref52] Piqueras J. Chassard C. Callon C. Rifa E. Theil S. Lebecque A. . (2021). Lactic starter dose shapes *S. aureus* and STEC O26:H11 growth, and bacterial community patterns in raw milk uncooked pressed cheeses. Microorganisms 9:1081. doi: 10.3390/microorganisms9051081, 34069983 PMC8157849

[ref53] Quigley L. OʼSullivan O. Beresford T. P. Ross R. P. Fitzgerald G. F. Cotter P. D. (2012). High-throughput sequencing for detection of subpopulations of bacteria not previously associated with artisanal cheeses. Appl. Environ. Microbiol. 78, 5717–5723. doi: 10.1128/AEM.00918-12 22685131 PMC3406138

[ref54] Rampanti G. Ferrocino I. Harasym J. Foligni R. Cardinali F. Orkusz A. . (2022). Queijo Serra da Estrela PDO cheese: investigation into its morpho-textural traits, microbiota, and volatilome. Foods 12:169. doi: 10.3390/foods12010169, 36613385 PMC9818377

[ref56] Richoux R. Maillard M. B. Kerjean J. R. Lortal S. Thierry A. (2008). Enhancement of ethyl ester and flavour formation in Swiss cheese by ethanol addition. Int. Dairy J. 18, 1140–1145. doi: 10.1007/s13594-016-0289-y

[ref57] Rocha R. Vaz Vello M. Santos J. Fernandes P. (2021). Serra da Estrela PDO cheese microbiome as revealed by next generation sequencing. Microorganisms 9:2007. doi: 10.3390/microorganisms9102007 34683326 PMC8537266

[ref58] Settanni L. Moschetti G. (2010). Non-starter lactic acid bacteria used to improve cheese quality and provide health benefits. Food Microbiol. 27, 691–697. doi: 10.1016/j.fm.2010.05.023, 20630311

[ref59] Spearman C. (1904). The proof and measurement of association between two things. Am. J. Psychol. 15, 72–101. doi: 10.2307/1412159 3322052

[ref1103] Štefániková J. Ducková V. Miškeje M. Kačániová M. Čanigová M. (2020). The impact of different factors on the quality and volatile organic compounds profile in “Bryndza” cheese. Foods 9, 1195. doi: 10.3390/foods9091195 32872403 PMC7555437

[ref60] Tormo J. Barral J. 2025. Accidents de fromagerie. Institut de l’Elevage, Lyon, France. Available online at: http://www.accident-fromagerie.fr/spip.php (Accessed July 4, 2025).

[ref1101] Unno R. Suzuki T. Matsutani M. Ishikawa M. (2021). Evaluation of the relationships between microbiota and metabolites in soft-type ripened cheese using an integrated omics approach. Front. Microbiol. 12, 681185. doi: 10.3389/fmicb.2021.681185 34168634 PMC8219077

[ref61] Walsh A. M. Macori G. Kilcawley K. N. Cotter P. D. (2020). Meta-analysis of cheese microbiotas highlights contributions to multiple aspects of quality. Nat. Foods 1, 500–510. doi: 10.1038/s43016-020-0129-3 37128079

[ref62] Wang Y. Qian P. Y. (2009). Conservative fragments in bacterial 16S rRNA genes and primer design for 16S ribosomal DNA amplicons in metagenomic studies. PLoS One 4:e7401. doi: 10.1371/journal.pone.0007401 19816594 PMC2754607

[ref63] Wang B. Wang J. XuL L. Y. Zhang J. H. Ai N. S. Cao Y. P. (2020). Characterization of key odorants in kurut with aroma recombination and omission studies. J. Dairy Sci. 103, 4164–4173. doi: 10.3168/jds.2019-17521 32173016

[ref64] Wang J. Yang Z. J. Wang Y. D. Cao Y. P. Wang B. Liu Y. (2021). The key aroma compounds and sensory characteristics of commercial cheddar cheeses. J. Dairy Sci. 104, 7555–7571. doi: 10.3168/jds.2020-19992, 33814151

[ref65] White T. J. Bruns T. Lee S. Taylor J. (1990). “Amplification and direct sequencing of fungal ribosomal RNA genes for phylogenetics” in PCR protocols: A guide to methods and applications. eds. Innis M. A. Gelfand D. H. Sninsky J. J. White T. J. (San Diego, CA, USA: Academic Press), 315–322. doi: 10.1016/B978-0-12-372180-8.50042-1

[ref66] Yeluri Jonnala B. R. McSweeney P. L. H. Sheehan J. J. Cotter P. D. (2018). Sequencing of the cheese microbiome and its relevance to industry. Front. Microbiol. 9:1020. doi: 10.3389/fmicb.2018.01020, 29875744 PMC5974213

