# Supplementary material for: Metataxonomic profiling of microbial communities and metabolic analyses of the traditional Spanish raw cow’s milk cheese ‘Casín’ from manufacture to ripening
Source: Front Microbiol. 2025 Dec 16;16:1722502. doi: 10.3389/fmicb.2025.1722502 (PMC12748158; doi:10.3389/fmicb.2025.1722502)
Supplement: Supplementary file 2 [file Supplementary_file_2.docx]

**Supplementary Table S2.-** Relative abundance of volatile organic compounds (VOC) detected in the Casín cheese samples of this study.

| **Class/compound** | **Cheese batch** | | | | | | | | | | | | | | | | | |
| --- | --- | --- | --- | --- | --- | --- | --- | --- | --- | --- | --- | --- | --- | --- | --- | --- | --- | --- |
|  | **A** | | | | | | **B** | | | | | | **C** | | | | | |
|  | **C** | **3 d** | **7 d** | **15 d** | **30 d** | **60 d** | **C** | **3 d** | **7 d** | **15 d** | **30 d** | **60 d** | **C** | **3 d** | **7 d** | **15 d** | **30 d** | **60 d** |
|  |  |  |  |  |  |  |  |  |  |  |  |  |  |  |  |  |  |  |
| **Acids** |  |  |  |  |  |  |  |  |  |  |  |  |  |  |  |  |  |  |
| Acetic acid | 30.8 | 276.7 | 390.4 | 216.3 | 261.3 | 196.1 | 3.6 | 156 | 134 | 145.9 | 186.7 | 155.3 | 9.5 | 268.1 | 104.9 | 115.9 | 108.8 | 127.7 |
| 9-Decenoic acid | 22.4 | 9.6 | 29 | 9.9 | 121.5 | 35.7 | 27.3 | - | 28.5 | 76 | 85.9 | 77.8 | 7.5 | 1.6 | 24.1 | 166.3 | 170.6 | 158.3 |
| Benzoic acid | - | 21.3 | - | 7.3 | - | 8.2 |  | 4.5 | - | - | - | - | - | 4.2 | - | - | - | - |
| Butanoic acid | 771.6 | 975.4 | 1652 | 1114 | 2437 | 2248 | 755.9 | 183.7 | 1317 | 2129 | 2287 | 2673 | 341.4 | 420.2 | 1850 | 3304 | 3269 | 3394 |
| Butanoic acid, 3-methyl | - | 12 | 100.4 | 60.7 | 52.9 | 59.4 | - | 8.8 | 51.5 | 61 | 155.6 | 117.6 | - | 4 | 49 | 18 | 17.5 | 17 |
| Dodecanoic acid | 23.9 | 13.1 | 23.4 | 5.4 | 104.1 | 17.1 | 17.2 | - | 24.1 | 51.7 | 57.9 | 48.2 | 3.7 | 2.5 | 16.1 | 142 | 130.2 | 138.2 |
| Heptanoic acid | 13.3 | 7.8 | 21 | 7.1 | 96.1 | 29.8 | 57.3 | 2.5 | 80 | 72.8 | 84.2 | 76.5 | 21.9 | 5.2 | 97.2 | 212.1 | 298.4 | 272 |
| Hexanoic acid | 958.2 | 870.2 | 1498 | 935.7 | 2664 | 1546 | 1097 | 191.9 | 1290 | 2108 | 2291 | 2276 | 632.4 | 376.5 | 1808 | 3267 | 3251 | 3128 |
| Hexanoic acid, 4-methyl | - | - | - | - | - | - | - | - | - | - | - | - | - | - | - | - | 9.4 | 9.4 |
| n-Decanoic acid | 228.7 | 132.2 | 254.1 | 92.3 | 644.9 | 277.6 | 235.6 | 27 | 265.3 | 469.1 | 495.6 | 508.8 | 90.2 | 29.1 | 209.3 | 798.9 | 817.8 | 797 |
| Nonanoic acid | 5.2 | 2.9 | 9.2 | 13.5 | 22.1 | 20 | 31.2 | 5.3 | 22.5 | 22.5 | 20.5 | 27.4 | 17.4 | 3.3 | 14.7 | 83 | 92.8 | 86.7 |
| Octanoic acid | 506.3 | 256.3 | 560.4 | 316.6 | 1304 | 728 | 642.1 | 61.2 | 643.5 | 949.4 | 1030 | 1028 | 409.6 | 99.7 | 649.4 | 1422 | 1440 | 1367 |
| Pentanoic acid | 17.5 | 22 | 66.8 | 23.3 | 133.6 | 77.4 | 45.8 | 3 | 93.6 | 115.6 | 131.8 | 139.5 | 9.9 | 12.5 | 206.9 | 379.9 | 363.6 | 364.2 |
| Propanoic acid | - | 4.3 | 86.6 | 15 | 30 | 39.5 | 0.8 | - | 6.1 | 9.6 | 21.7 | 30.5 | - | - | 11.3 | 33.9 | 36.6 | 40.9 |
| Propanoic acid, 2-methyl | - | 3.2 | 25.4 | 13.4 | 10.1 | 20.7 | - | 1.7 | 13 | 49.7 | 56.5 | 37.3 | - | - | 14.2 | - | - | - |
| Tetradecanoic acid | 3.6 | - | - | - | 20.3 | - | - | - | - | - | - | - | - | - | - | 24 | 20.3 | 22.9 |
| Undecanoic acid | ^-^ | - | - | - | - | - | - | - | - | - | - | - | - | - | - | 14 | 12.6 | 13.1 |
|  |  |  |  |  |  |  |  |  |  |  |  |  |  |  |  |  |  |  |
| **Aldehydes** |  |  |  |  |  |  |  |  |  |  |  |  |  |  |  |  |  |  |
| 2,4-Heptadienal, (E,E) | - | - | - | - | - | - | - | 0.7 | - | - | - | - | - | - | - | - | - | - |
| 2-Nonenal, (Z) | - | - | - | - | - | - | - | 0.1 | - | - | - | - | - | - | - | - | - | - |
| 2-Octenal, (E) | - | - | - | - | - | - | - | 1.9 | - | - | - | - | - | - | - | - | - | - |
| Benzaldehyde | - | 25.5 | 5.5 | 15.2 | - | - | - | 11.2 | 9.5 | 8.9 | 4.7 | 4 | 2.1 | 3.6 | - | - | - | - |
| Nonanal | - | - | - | - | - | - | - | 1.5 | - | - | - | 6 | - | 4 | - | - | - | - |
|  |  |  |  |  |  |  |  |  |  |  |  |  |  |  |  |  |  |  |
| **Alcohols** |  |  |  |  |  |  |  |  |  |  |  |  |  |  |  |  |  |  |
| 1,3-Dioxolane-2,2-diethanol | - | - | - | - | - | - | - | - | - | 3.3 | - | - | - | - | - | - | - | - |
| 1-Butanol | - | - | 116 | - | - | - | - | - | - | - | - | - | - | - | - | - | - | - |
| 1-Butanol, 3-methyl | - | 156.9 | 107.2 | 109.6 | - | - | - | 40.8 | 31.1 | - | 21.4 | - | - | 20.5 | - | - | - | - |
| 1-Hexanol | - | - | 36.9 | - | - | - | - | 21 | - | - | - | - | - | 105.5 | 95 | - | - | - |
| 2-Heptanol, (S) | - | - | 19.1 | - | - | 42.4 | - | - | - | - | 142.1 | - | - | - | - | - | - | - |
| 2-Nonanol | - | - | - | - | 13.9 | 24.4 | - | - | - | - | 52.4 | 59.9 | - | - | - | - | - | - |
| 2-Pentanol | - | - | - | - | - | - | - | - | - | - | - | 7.6 | - | 10.5 | - | - | - | - |
| Benzyl alcohol | - | 5.3 | 17.2 | - | 8.1 | 11 | - | 4.9 | 4.9 | 4.6 | 12 | 21.3 | - | 33.3 | 54.4 | 13.9 | 16.3 | 17.2 |
| Phenylethyl Alcohol | - | 33.8 | 41.9 | 34.4 | 14 | 7.7 | - | - | 17.7 | 25.6 | - | 26.6 | - | - | 7.7 | - | - | - |
|  |  |  |  |  |  |  |  |  |  |  |  |  |  |  |  |  |  |  |
| **Esters** |  |  |  |  |  |  |  |  |  |  |  |  |  |  |  |  |  |  |
| 1,2-Propanediol dibutyrate | - | - | - | - | - | - | - | - | - | 9.1 | 11.6 | 12.2 | - | - | - | - | - | - |
| Acetic acid, 2-phenylethylester | - | 35.7 | 36.8 | 20.9 | 25.2 | 10.7 | - | - | - | - | - | - | - | - | - | - | - | - |
| Acetic acid, heptyl ester | - | - | - | - | - | - | - | - | - | - | 6 | 12.4 | - | - | - | - | - | - |
| Butanoic acid, 1-methylbutyl ester | - | - | - | - | - | - | - | - | - | - | 68.7 | 60.6 | - | - | - | - | - | - |
| Butanoic acid, 3-methylbutyl ester | - | - | 21.2 | 9.8 | 13.9 | 14 | - | - | 3.4 | 90.3 | 48.8 | 39.8 | - | - | 14.5 | - | - | - |
| Butanoic acid, butyl ester | - | - | - | 11.4 | 83.1 | 61.7 | - | - | - | 95.5 | 136.5 | 108.7 | - | - | 15 | 98.4 | - | - |
| Butanoic acid, ethyl ester | - | - | - | 276.4 | - | - | - | - | - | 32.8 | 43.9 | - | - | 60.1 | 151.7 | - | 128.4 | - |
| Butanoic acid, hexyl ester | - | - | - | - | - | - | - | - | - | 4.4 | 4 | 3.5 | - | - | 32 | - | - | - |
| Butanoic acid, pentyl ester | - | - | - | 44.1 | - | - | - | - | - | - | - | - | - | - | - | - | - | - |
| Butanoic acid, propyl ester | - | - | 219.3 | - | - | 82.9 | - | - | - | - | - | - | - | - | - | - | - | - |
| Butyl 3-methylpentanoate | - | - | - | - | - | - | - | - | - | - | - | - | - | - | 8.8 | - | - | - |
| Butyl caprate | - | - | - | - | - | - | - | - | - | 42.4 | 54.1 | 44.1 | - | - | - | - | - | - |
| Decanoic acid, ethyl ester | - | - | - | - | - | - | - | - | - | - | - | - | - | - | - | 477.6 | 400.9 | 252.3 |
| Decanoic acid, methyl ester | 15.6 | - | - | - | - | - | 9.9 | - | 10.4 | 7.2 | 11.2 | 11.6 | 12.7 | - | - | 51.9 | 31.2 | 18.8 |
| Decanoic acid, propyl ester | - | - | 30.5 | - | 14.5 | 13.1 | - | - | - | - | - | - | - | - | - | - | - | - |
| Ethyl 9-decenoate | - | - | 4.1 | 2.7 | 8.9 | 5.7 | - | - | 6.1 | 16.1 | 15.3 | 7.3 | - | - | 4.7 | 55 | 31.6 | 22.2 |
| Heptanoic acid, ethyl ester | - | - | - | - | - | - | - | - | 6.8 | 45.1 | - | - | - | - | 23.1 | 70 | 52.6 | 38.7 |
| Hexanoic acid, 2-methylpropyl ester | - | - | - | - | - | - | - | - | 28.3 | 6 | 303.2 | 189.1 | - | - | - | - | - | - |
| Hexanoic acid, 3-pentylester | - | - | - | - | 49.6 | 7 | - | - | - | 23.3 | 74.8 | 92 | - | - | - | - | - | - |
| Hexanoic acid, butyl ester | - | - | 17.2 | - | 15 | 20.4 | - | - | - | 295.9 | 5.6 | 5.9 | - | - | - | - | - | - |
| Hexanoic acid, ethyl ester | 47.6 | 125.4 | 859 | 554.8 | 231.4 | 571.6 | 27.3 | 8.3 | 203 | 513 | 549.2 | 379.9 | 24 | 23.5 | 683.9 | 1106 | 807.2 | 517.4 |
| Hexanoic acid, methyl ester | - | - | 9.9 | 6.7 | - | - | 63.2 | - | 69 | - | - | - | 40.1 | - | 251.5 | 80.5 | - | - |
| Hexanoic acid, pentyl ester | - | - | - | - | 12.1 | 6.6 | - | - | - | 14.2 | 16.9 | 16.5 | - | - | - | - | - | - |
| Hexanoic acid, propyl ester | - | - | 230.8 | - | 88.7 | 90.6 | - | - | - | - | - | - | - | - | - | - | - | - |
| n-Capric acid isopropyl ester | - | - | - | - | - | - | - | - | - | 74 | - | - | - | - | - | - | - | - |
| n-Caprylic acid isobutyl ester | - | - | - | - | - | - | - | - | - | - | 58.2 | - | - | - | - | - | - | - |
| n-Octanoic acid isopropyl ester | - | - | - | - | 16.7 | - | - | - | - | - | 8.7 | 9.5 | - | - | - | - | - | - |
| Nonanoic acid, ethyl ester | - | - | - | - | - | - | - | - | 3.6 | 17.3 | - | - | - | - | - | 20 | 11.9 | - |
| Octanoic acid, 2-butylester | - | - | - | - | - | - | - | - | - | - | - | 32.2 | - | - | - | - | - | - |
| Octanoic acid, ethyl ester | 23.4 | 22.4 | 177.8 | 80.3 | 266.7 | 290.6 | 9.4 | 1.9 | 188 | 275.5 | 277.5 | 156.1 | 12.3 | 5.4 | 221.4 | 887.2 | 594.6 | 481.9 |
| Octanoic acid, methyl ester | - | - | - | - | - | - | 27.5 | 0.1 | - | - | 0.5 | - | 23.2 | - | 93.5 | 185.8 | - | - |
| Pentanoic acid,5-hydroxy,2,4di-butylphenyl esters | - | - | - | 2 | - | - | 2.7 | - | - | - | - | - | 2.4 | - | - | - | - | - |
| Propanoic acid, 2-amino-3-hydroxy-, ethyl ester | - | - | 16.8 | - | - | - | - | - | - | - | - | - | - | - | - | - | - | - |
| Propanoic acid, 2-methyl-, ethyl ester | - | - | 4.8 | - | - | 26.2 | - | - | - | - | - | 42.6 | - | - | 7.4 | - | - | - |
| Propyl octanoate | - | - | 43.3 | - | 48.2 | 31.6 | - | - | 12.9 | - | - | - | - | - | - | - | - | - |
| Tetradecanoic acid, ethyl ester | - | - | - | - | - | - | - | - | - | 2.7 | - | - | - | - | - | - | - | - |
| Tetrahydro-2-furanylpropanoic acid | - | - | 8.3 | - | 59.8 | - | - | - | - | - | - | - | - | - | - | - | - | - |
|  |  |  |  |  |  |  |  |  |  |  |  |  |  |  |  |  |  |  |
| **Ketones** |  |  |  |  |  |  |  |  |  |  |  |  |  |  |  |  |  |  |
| 2-Decanone | - | - | - | - | 10.3 | - | - | - | - | - | 6.5 | 13.2 | - | - | - | - | - | - |
| 2-Heptanone | 109.5 | 24.7 | - | 13.9 | 934.9 | 30.8 | 36.6 | 25.7 | 69.2 | 340.6 | 1075 | 1224 | 6.9 | 27.3 | - | 180.3 | 214.8 | 168.5 |
| 2-Hexanone, 4-methyl- | - | - | - | - | - | - | 17.1 | 0.7 | - | - | - | - | - | - | - | - | - | - |
| 2-Nonanone | 51.6 | 8.9 | - | 6.7 | 993 | 57.6 | - | 8.3 | 90.1 | 313.5 | 931 | 1458 | - | 5.9 | - | 50.7 | 203.7 | 175.8 |
| 2-Octanone | - | - | - | - | 24.9 | - | - | 0.1 | - | 9.8 | 65.9 | 122.7 | - | - | - | - | - | - |
| 2-Pentanone | - | - | - | - | 539.9 | - | - | - | - | - | - | - | - | - | - | 17 | 38.1 | - |
| 2-Undecanone | 3.5 | - | - | - | 182.4 | 18.2 | - | - | 6.4 | 36.3 | 54.8 | 75.8 | - | - | - | 53.4 | 48.7 | 40.6 |
| 5-Hepten-2-one | - | - | - | - | - | - |  | - | - | - | - | 33.2 | - | - | - | - | - | - |
| 8-Nonen-2-one | 3.9 | - | - | - | 255.6 | - | - | 0.5 | 4.9 | 39.3 | 242.9 | 507.1 | - | - | - | - | - | - |
| Acetoin | - | 98.9 | 7.9 | 29 | 32.9 | 18.5 | 22.6 | 311.7 | 547.4 | 61.4 | 48.1 | 52.2 | 42.4 | 76.5 | 28.4 | - | - | - |
|  |  |  |  |  |  |  |  |  |  |  |  |  |  |  |  |  |  |  |
| **Lactones** |  |  |  |  |  |  |  |  |  |  |  |  |  |  |  |  |  |  |
| 2(3H)-Furanone, 5-butyldihydro | - | - | - | - | - | - | - | - | - | - | - | - | - | - | 9.5 | 11 | 9.9 | 9.3 |
| 2(3H)-Furanone, 5-ethyldihydro | - | - | - | - | - | - | - | - | - | - | - | - | - | - | - | - | 4.7 | 9.6 |
| 2H-Pyran-2-one,tetrahydro-6-nonyl | - | - | - | - | - | - | - | - |  | - | - | - | - | 1 | 2.3 | 6.6 | - | - |
| 2H-Pyran-2-one,tetrahydro-6-pentyl | 4.9 | 5.1 | 4.6 | 2.2 | - | - | - | 3.5 | 4.3 | - | - | - | 3 | 3.6 | - | - | - | - |
| 2H-Pyran-2-one,tetrahydro-6-propyl | - | - | - | - | - | - | - | 1.3 |  | - | - | - | - | 0.6 | - | - | - | - |
| 2H-Pyran-2-one,tetrahydro-6-octyl | - | - | - | - | - | - | 2.3 | - | - | 1.9 | - | - | - | - | - | - | - | - |
|  |  |  |  |  |  |  |  |  |  |  |  |  |  |  |  |  |  |  |
| **Phenols** |  |  |  |  |  |  |  |  |  |  |  |  |  |  |  |  |  |  |
| 2,4-Di-tert-butylphenol | - | - | - | - | - | - | - | 1.4 | - | - | - | - | - | - | - | - | - | - |
| p-Cresol | - | - | 17.3 | - | 15.1 | - | - | - | 3.6 | 7.4 | 8.4 | 11.1 | - | - | - | - | - | - |
| Phenol | - | - | - | - | - | - | - | - | 7.7 | - | - | 6.5 | - | - | - | - | - | - |
| Phenol, 3-methyl | - | - | 15.7 | - | 16.1 | - | - | - | - | - | - | - | - | - | - | - | - | - |
|  |  |  |  |  |  |  |  |  |  |  |  |  |  |  |  |  |  |  |
| **Others** |  |  |  |  |  |  |  |  |  |  |  |  |  |  |  |  |  |  |
| 1-Methoxy-3-(2-hydroxyethyl)nonane | - | - | - | - | - | - | - | - | - | - | 15.9 | 21.1 | - | - | - | - | - | - |
| Caprolactam | - | - | - | 2.2 | - | 1.9 | 2.3 | 1.4 | - | - | - | - | 1.2 | 1.6 | 4.8 | - | - | - |
| Dimethyl sulfone | 9.1 | 5.6 | 11.2 | - | 5.3 | 3.8 | - | - | - | - | - | - | - | - | - | - | - | - |
| Heptanediamide, N,N'-di-benzoyloxy | - | - | 24.3 | - | 18.6 | - | - | - | - | 7.6 | 8.1 | 7.4 | - | - | 3.7 | - | - | - |
| Mesitylene | - | - | - | - | - | - | - | - | - | 4.4 | - | - | - | - | - | - | - | - |
| Phenoxy-2-chloropropane | - | - | - | - | - | - | - | - | - | 7.8 | 0.1 | - | - | - | - | - | - | - |
|  |  |  |  |  |  |  |  |  |  |  |  |  |  |  |  |  |  |  |

Key of colours: Green, majority compounds; orange, subdominant compounds; pink, differential compounds.
